# The plasma lipidome varies with the severity of metabolic dysfunction-associated steatotic liver disease

**DOI:** 10.1186/s12944-024-02380-x

**Published:** 2024-12-18

**Authors:** Clément J. F. Heymann, Anne Linde Mak, Adriaan G. Holleboom, Joanne Verheij, Ronit Shiri-Sverdlov, Saskia W. C. van Mil, Maarten E. Tushuizen, Ger H. Koek, Aldo Grefhorst

**Affiliations:** 1 Department of Experimental Vascular Medicine, Amsterdam University Medical Centers, Location AMC, Amsterdam, The Netherlands; 2 Department of Vascular Medicine, Amsterdam University Medical Centers, Location AMC, Amsterdam, The Netherlands; 3 Department of Pathology, Amsterdam University Medical Centers, Location AMC, Amsterdam, The Netherlands; 4 Department of Genetics and Cell Biology, Maastricht University Medical Centre, Maastricht, The Netherlands; 5 School of Nutrition and Translational Research in Metabolism (NUTRIM), Maastricht University, Maastricht, The Netherlands; 6 Center for Molecular Medicine, University Medical Center Utrecht and Utrecht University, Utrecht, The Netherlands; 7 Department of Gastroenterology and Hepatology, Leiden University Medical Centre, Leiden, The Netherlands; 8 Department of Internal Medicine, Division of Gastroenterology and Hepatology, Maastricht University Medical Centre, Maastricht, The Netherlands

**Keywords:** Lipidomics, Lipids, Lipoproteins, Liver, MASLD, Phospholipids/phosphatidylcholine

## Abstract

**Background:**

Metabolic dysfunction-associated steatotic liver disease (MASLD) is closely associated with many aspects of disturbed metabolic health. MASLD encompasses a wide spectrum of liver diseases, ranging from isolated steatosis to metabolic dysfunction-associated steatohepatitis (MASH), up to fibrosis, cirrhosis, and ultimately hepatocellular carcinoma. Limited noninvasive diagnostic tools are currently available to distinguish the various stages of MASLD and as such liver biopsy remains the gold standard for MASLD diagnostics. We aimed to explore whether the plasma lipidome and its variations can serve as a biomarker for MASLD stages.

**Methods:**

We investigated the plasma lipidome of 7 MASLD-free subjects and 32 individuals with MASLD, of whom 11 had MASH based on biopsy scoring.

**Results:**

Compared with the MASLD-free subjects, individuals with MASLD had higher plasma concentrations of sphingolipids, glycerolipids, and glycerophospholipids. Only plasma concentrations of ceramide-1-phosphate C1P(d45:1) and phosphatidylcholine PC(O-36:3), PC(O-38:3), and PC(36:2) differed significantly between presence of MASH in individuals with MASLD. Of these lipids, the first three have a very low relative plasma abundance, thus only PC(36:2) might serve as a biomarker with higher plasma concentrations in MASLD individuals without MASH compared to those with MASH.

**Conclusions:**

Plasma lipids hold promise as biomarkers of MASLD stages, whereas plasma PC(36:2) concentrations would be able to distinguish individuals with MASH from those with MASLD without MASH.

**Supplementary Information:**

The online version contains supplementary material available at 10.1186/s12944-024-02380-x.

## Background

Over the last decades, high caloric intake and sedentary lifestyle have driven obesity, type 2 diabetes mellitus (T2DM) and progressively metabolic dysfunction-associated steatotic liver disease (MASLD). MASLD is a rather recent term, replacing non-alcoholic fatty liver disease (NAFLD) [1] and, in general, subjects who were diagnosed as having NAFLD will now be diagnosed with MASLD [2]. With a global prevalence of over 25%, MASLD has become the most prevalent chronic liver disease worldwide [3–6]. The disease spectrum of MASLD is characterized by a worsening scale of features, ranging from isolated steatosis, metabolic dysfunction-associated steatohepatitis (MASH), up to fibrosis, and in certain cases cirrhosis or hepatocellular carcinoma (HCC) [7, 8]. In addition to liver-related complications, progressive MASLD may also drive atherosclerotic cardiovascular disease, most likely through changes in plasma lipids [9]. Given its close epidemiological and pathophysiological links with obesity, T2DM, hyperlipidemia and atherosclerosis, MASLD can be considered the hepatic representation of the metabolic syndrome [10, 11].

Key to the development of MASLD and therefore MASH is the excess accumulation of triglycerides (TGs) in the liver. In theory, this excess accumulation can be caused by (a combination of) uptake of circulating fatty acids, enhanced hepatic de novo lipogenesis, reduced secretion of TGs in very low density lipoprotein (VLDL) particles, and a disturbed fatty acid oxidation (FAO) [12]. Of these four pathways, the increased fatty acid influx has been shown to be the predominant cause of MASLD [13], likely due to insulin resistance (IR) that will result in elevated lipolysis in peripheral adipose tissues and thus an enhanced flux of fatty acids to the liver. This aggregation of lipids in hepatocytes overwhelms the physiological healthy hepatic capacity to store, secrete and oxidize lipids, resulting in lipotoxicity within the steatotic liver [11].

Currently, no accurate diagnostic tools are available to determine the presence of MASLD or detect progression to MASH. Liver biopsy remains the gold standard for MASLD diagnostics to determine fibrotic stages, inflammation and ballooning, but this method comes with obvious drawbacks due to its invasive nature [14]. Thus, there is a need to develop non-invasive biomarkers to differentiate MASLD stages. Although some have already been developed [15], these biomarkers lack in sensitivity and are predominantly aimed at determining fibrosis and thus ignore other underlying mechanisms of MASH such as lipotoxicity. Studying the plasma lipid profiles in an unbiased way by lipidomics might aim to uncover a lipid biomarker of MASLD stages. Plasma lipidomics in MASLD has been performed before, as recently reviewed by Beland-Bonenfant et al. [16] and Musso et al. [17]. Although some lipid species such as lysophoshatidylcholine (LPC) and diacylglycerol (DG) have been proposed to specifically mediate hepatic lipotoxicity [18–22] and might be suitable for the identification of MASH in individuals with MASLD, detailed exploration of the association of the plasma lipids with MASLD stages, i.e. MASH progression, is lacking.

In order to identify plasma lipid signatures of disease severity along the MASLD spectrum, i.e., depending on different methods of scoring MASLD with or without MASH, we used a lipidomic platform to identify and semi-quantify over 1600 lipids in the plasma of 39 individuals with histologically characterized MASLD of whom some had MASH. With this, we found that plasma phosphatidylcholine PC(29:1) is elevated in subjects with MASLD independent of MASH and that plasma PC(36:2) concentrations might be suitable to distinguish MASLD individuals with MASH from those without MASH.

## Materials and methods

### Study design, population and MASLD/MASH grading

The cohort of individuals with MASLD analyzed in this study, consisting of 32 individuals with MASLD and 7 MASLD-free subjects (Table S1), originated from two academic medical centers in The Netherlands, namely the Amsterdam University Medical Centers (AUMC) and the Maastricht University Medical Center (MUMC+). The protocol was reviewed and approved by the institutional review boards of the medical centers (AMC METC 2013_207 and METC 142074 for the AUMC and MUMC+, respectively) and abide the declaration of Helsinki principles. All participants provided written informed consent.

The individuals were included between 1/1/2014 and 31/12/2017 and between 1/2/2017 and 31/1/2019 in the AUMC and MUMC+, respectively. All individuals were Caucasian, overweight, treatment-naïve subjects with hepatic steatosis on ultrasound (see for detailed criteria Witjes et al. [23]) that abide to the new cardiometabolic MASLD criteria. The histological status of each subject was determined by tandem reading by qualified liver pathologists.

Percutaneous ultrasound-guided liver biopsies were obtained during clinical workup or during bariatric surgery were performed by either an interventional radiologist or a hepatologist according to local standard procedure. Biopsies were stained with a hematoxylin and eosin stain and a Sirius Red stain. All histologic specimens were scored by liver pathologists blinded to all other data. Steatosis grade (0–3) depends on the percentage of hepatocytes containing large and medium-sized intracytoplasmic lipid droplets: in grade 0 < 5% of the hepatocytes contain lipid droplets; in grade 1 5–33%; in grade 2 34–66%; and in grade 3 > 67% of hepatocytes contain lipid droplets. Fibrosis was scored according to the NASH Clinical Research Network (CRN) scoring system [24]. In stage 0 there is no detectable fibrosis. In stage 1 there is perisinusoidal (in zone 3) or periportal (in zone 1) fibrosis, stage 2 encompasses fibrosis in both the perisinusoidal and periportal zones but without bridging while there is bridging fibrosis in stage 3. Stage 4 or cirrhosis is present in case of extensive bridging fibrosis. The NAFLD Activity Score (NAS) is the sum of the steatosis grade, lobular inflammation and the ballooning of the hepatocytes [25].

An individual was considered to have MASLD when lipid droplets were present in at least 5% of the hepatocytes. MASH was diagnosed in those individuals with MASLD when hepatocellular ballooning grade was ≥ 1 with a lobular inflammation grade ≥ 1.

### Lipidomics

Lipidomics was performed as previously described [26, 27], with minor adjustments. In brief, 25 µl of plasma sample was added to a 2 ml tube after which the following amounts of internal standards dissolved in methanol: chloroform (1:1 v/v) were added: 0.2 nmol bis(monoacylglycero)phosphate BMP(14:0/14:0), 0.127 nmol C1P(d18:1/12:0), 2 nmol D7-CE(16:0), 0.118 nmol Cer(d18:1/12:0), 0.130 nmol Cer(d18:1/25:0), 0.1 nmol cardiolipin CL(14:0/14:0/14:0/14:0), 0.5 nmol DG(14:0/14:0), 0.126 nmol glucose-ceramide Glc-Cer(d18:1/12:0), 0.129 nmol lactose-ceramide LacCer(d18:1/12:0), 0.1 nmol LPA(14:0), 0.5 nmol LPC(14:0), 0.1 nmol LPE(14:0), 0.02 nmol lysophosphatidylglycerol LPG(14:0), 0.5 nmol PA(14:0/14:0), 2 nmol PC(14:0/14:0), 0.5 nmol PE(14:0/14:0), 0.1 nmol phosphatidyl-glycerol PG(14:0/14:0), 0.5 nmol PI(8:0/8:0), 5 nmol PS(14:0/14:0), 0.124 nmol sphinganine-1-phosphate S1P(d17:0), 0.125 nmol S1P(d17:1), 2.129 nmol ceramidephosphocholine SM(d18:1/12:0), 0.125 nmol SPH(d17:0), 0.125 nmol SPH(d17:1), and 0.5 nmol TG(14:0/14:0/14:0). When not explained here, abbreviations are explained in Table S2. Next, 1.5 ml methanol: chloroform (1:1 v/v) was added and the samples were thoroughly mixed. The samples were centrifuged for 10 min at 14,000 rpm, the supernatant was transferred to a glass vial and evaporated under a stream of nitrogen at 60 °C. The residue was dissolved in 150 µl of methanol: chloroform (1:1 v/v) and the lipids were analyzed using a Thermo Scientific Ultimate 3000 binary HPLC coupled to a Q Exactive Plus Orbitrap mass spectrometer (MS).

For normal phase separation, 2 µl of the sample was injected onto a Phenomenex^®^ LUNA column (5 μm silica, 100 Å, 250 × 2 mm) that was kept at 25 °C. The mobile phase consisted of solvent A (methanol: water (85:15 v/v) with 0.0125% formic acid and 3.35 mM ammonia) and solvent B (chloroform: methanol (97:3 v/v) with 0.0125% formic acid). The flow rate was set at 0.3 ml/min and the percentage of solvent A was 10% for the first minute and was steadily increased until it reached 20% at 4 min, 85% at 12 min and 100% A at 12.1 min. The mobile phase was kept at 100% solvent A until 14 min and decreased to 10% solvent A at 14.1 min and kept at 10% until 15 min. For reversed phase separation, 5 µl of the sample was injected onto a Waters HSS T3 column (1.8 μm particle size, 150 × 2.1 mm) that was kept at 60 °C. The mobile phase consisted of solvent C (methanol: water (4:6 v/v) with 0.1% formic acid and 10 mM ammonia) and solvent D (methanol: isopropanol (1:9 v/v) with 0.1% formic acid and 10 mM ammonia). The flow rate was set at 0.4 ml/min and the percentage of solvent C was 100% at start but was steadily decreased until it reached 80% at 1 min and reach 0% at 16 min. The mobile phase was kept at 0% solvent C until 20 min and increased to 100% solvent C at 20.1 min and kept at 100% until 21 min.

### Bioinformatics for lipid identification

MS data were acquired using negative and positive ionization using continuous scanning over the range of m/z 150 to m/z 2000. These raw data were converted to mzXML format using MSConvert. The dataset was processed using an in-house developed lipidomics pipeline written in the R programming language 3 (R Foundation for Statistical Computing, Vienna, Austria, www.r-project.org). In brief, it consisted of the following steps: pre-processing using the R package XCMS with minor changes to some functions in order to better suit the Q Exactive data; notably, the definition of noise level in centWave was adjusted and the stepsize in fillPeaks; identification of metabolites using an in-house database of (phospho)lipids, with known internal standards indicating the position of most of the lipid clusters, matching m/z values within 3 ppm deviation; isotope correction to obtain deconvoluted intensities for overlapping peak groups; normalization on the intensity of the internal standard for lipid classes for which an internal standard was available and scaling on measured protein content per sample.

### Lipid species abbreviations

Each lipid class was abbreviated with a defined letter code. For instance, glycerophospholipids abbreviations (e.g. PC, PE) were used to refer to species with two radyl side chains, where the total lengths of the side chains and total number of double bonds were indicated within parentheses: Headgroup (Length of side chains : Number of double bonds). Corresponding species with only side chain were referred as Lyso-PL (e.g. LPC, LPE, etc.). Only the total mass of the lipids was recorded, not the exact composition of the localization of individual side chains. The actual structure of each lipid can be hypothesized based on known lipid biochemistry (e.g. PC(34:1) is most likely the sum of PC(16:1/18:0) and PC(18:1/16:0)).

### Statistical analyses

Analyses were done in R version 4.1.2 (2021-11-01). Spearman correlation analyses were performed using Past4 software and checked in R. In the lipidomics analysis, summary data for each lipid class was calculated by measuring the mean value of individual lipid species. Total lipid levels of individual lipid classes were obtained by summing up the mean values of all lipid subspecies together. The control group was compared to the different disease groups, using a One-way ANOVA, followed by a Tukey-Post Hoc test. A p-value of 0.05 or less was considered significant. Unless mentioned otherwise, p-values were corrected for multiple testing per lipid class by applying a Benjamini & Hochberg correction with FDR < 5%. Serum levels of individual lipids were correlated with the histological profiles of the subject (Steatosis / Fibrosis / NAS scores) by Spearman correlation analysis. Based on the uncorrected p-value, a top 30 of lipids correlating with each histological variable was drawn. Heatmaps were created for the different top 30 rankings by calculating the z-score of each individual samples in a row (metabolite) as follows: Z-score = (Value of individual sample – Mean of the row) / Standard deviation of the row.

The graphical representations provide an indication on how far the raw level of a given lipid is compared to the mean value of concerned lipid class. In heatmaps, the individuals were classified based on their histological status, i.e., inflammatory activity and fibrotic stage, to provide insights on fluctuations of lipid levels across disease severity. Finally, volcano plots comparing the serum lipid fold-changes of control subjects with serum fold-changes of individuals with mild/severe disease states were created. To do so, the entire cohort was redistributed in multiple subgroups based on the histological profiles. The control group was then compared to mild/severe steatosis groups, mild/severe fibrotic groups, and low/high NAS score groups.

## Results

### Characteristics of the included subjects

We included 39 subjects of whom, based on histological scoring, 7 did not have MASLD and 32 had MASLD. Of the latter group, 11 had MASH (Table 1 and Table S1). Apart from a higher average body mass index (BMI) in the MASLD-free subjects, and higher plasma gamma-glutamyl transferase (GGT), alanine aminotransferase (ALT) and aspartate aminotransferase (AST) concentrations in those with MASLD, the clinical characteristics did not differ between the groups. In general, the histological examination revealed that hepatic steatosis grades, fibrosis grades and NAS scores had a high heterogeneity within and between subgroups (Table 2).

**Table 1:** Clinical characteristics of the 39 included subjects

	MASLD-free	MASLD w/o MASH	MASLD w/ MASH
Age (years)	36 (30–50)	55 (48–58)	52 (53–56)
Weight (kg)	110 (107–115)	96 (90–111)	95 (88–108)
Height (cm)	169 (167–173)	181 (177–183) ^*^	173 (169–179)
BMI (kg/m^2^)	38.6 (38.5–39.3)	32.5 (30.0-34.5) ^*^	32.7 (30.9–33.7) ^*^
GGT (IU/l)	22.0 (15.0-23.5)	47.0 (31.0–64.0) ^*^	52.0 (46.0–85.0) ^*^
ALT (IU/l)	19.0 (18.5–27.5)	53.0 (37.0–66.0) ^*^	71.0 (45.5–81.0)
AST (IU/l)	19.0 (17.529.0)	37.0(30.0–39.0)	54.0 (41.5–108.0) ^*,#^
TC (mM)	4.1 (4.0-4.3)	5.4 (5.2-6.0) ^*^	5.1 (4.6-6.0)
LDL-c (mM)	2.7 (2.5–2.9)	3.5 (3.3–4.3)	2.8 (2.3–3.9)
HDL-c (mM)	1.0 (1.0-1.2)	1.2 (1.0-1.4)	1.2 (1.0-1.4)
TG (mM)	1.3 (1.0-1.5)	1.3 (1.1–1.4)	1.9 (1.4–3.5)
HbA1c (mmol/mol)	35.0 (33.0-38.5)	39.0 (34.0–40.0)	41.0 (36.5–54.5)
Glucose (mM)	5.4 (5.1–6.1)	5.9 (5.3–6.3)	6.4 (5.6–7.2)
T2DM, n (%)	1 (14.3)	0 (0.0)	3 (27.3)

Values are medians with interquartile range in parentheses. BMI, body mass index; GGT, gamma-glutamyl transferase; ALT, alanine aminotransferase; AST, aspartate aminotransferase; TC, total cholesterol; LDL-c, low density lipoprotein cholesterol; HDL-c, high density lipoprotein cholesterol; TG, triglycerides; HbA1c, Hemoglobin-A1c; T2DM, type 2 diabetes mellitus; *, *p* < 0.05 vs. healthy controls; #, *p* < 0.05 vs. MASLD without MASH (Kruskal-Wallis tests or Chi-square test)

**Table 2:** Histological liver characteristics of the 39 included subjects

	MASLD-free	MASLD w/o MASH	MASLD w/ MASH
Steatosis grade, n (0/1/2/3)	7/0/0/0	0/12/6/3 ^*^	0/3/6/2 ^*^
Fibrosis grade, n (0/1/2/3)	5/2/0/0	5/13/3/0 ^*^	1/3/2/5 ^*,#^
NAS score, n (0/1–2/3–4/5/6)	3/4/0/0	0/12/9/0 ^*^	0/0/7/4 ^*,#^
Inflammation, n (0/1/2)	3/4/0	1/19/1 ^*^	0/9/2 ^*^
Ballooning, n (0/1/2)	7/0/0	21/0/0	0/9/2 ^*,#^

*, *p* < 0.05 vs. healthy controls; #, *p* < 0.05 vs. MASLD without MASH (Chi-square test)

### Several lipids are correlated with steatosis grade

**Fig. 1:**
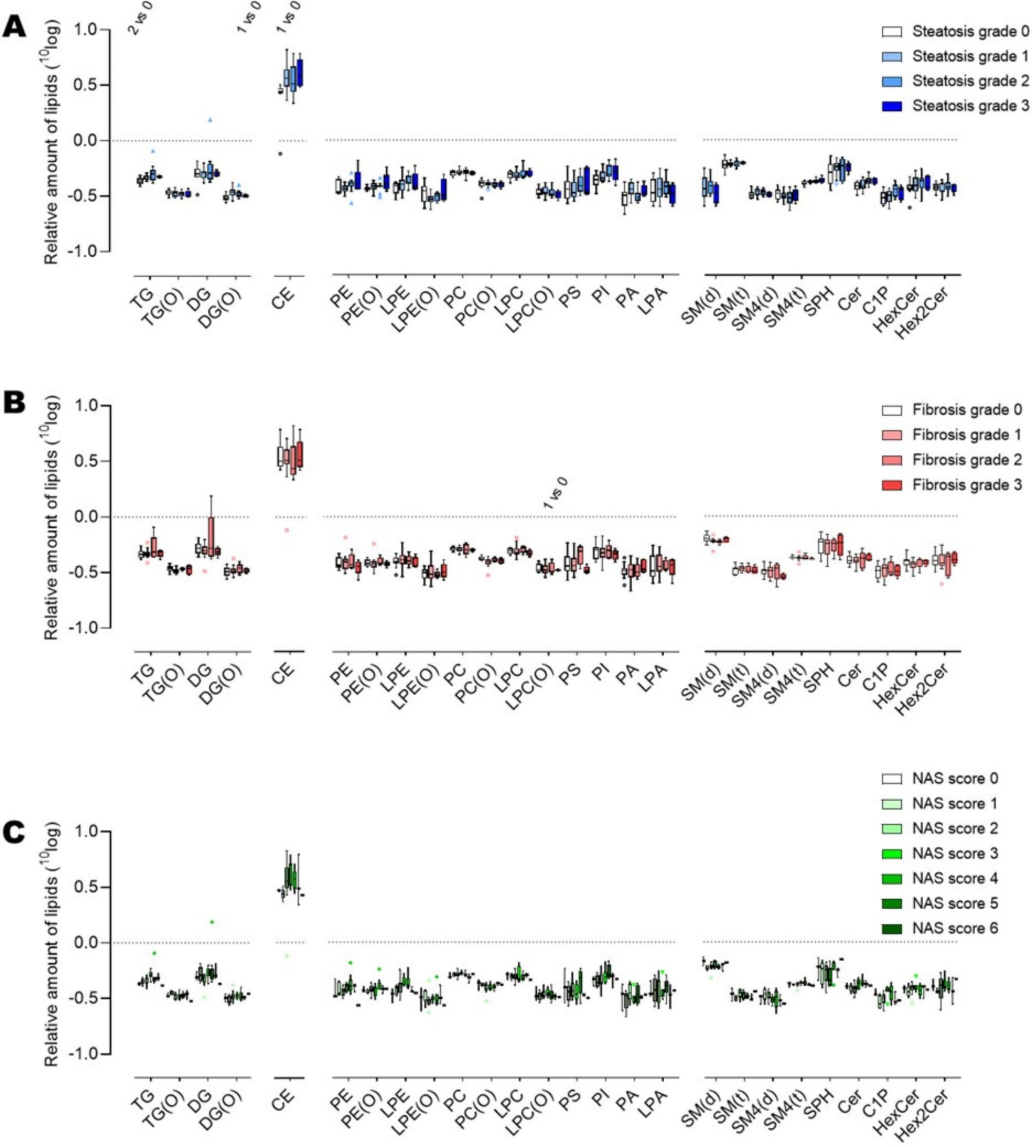
Relative abundance of plasma concentrations of the lipid classes in subjects with respect to their (**A**) hepatic steatosis grade, (**B**) fibrosis grade, and (**C**) NAS score. Data are shown as box plots. The inserted text mentions whether there is a significant difference (*p* < 0.05, Mann-Whitney U test) between the grades or scores for the specific lipid class

The plasma of the 39 subjects were used for lipidomic analysis in which 1625 lipids distributed over 17 different lipid categories (Table S2) were analyzed. When comparing the plasma lipid composition between subjects with various histological grades of MASLD (steatosis grade, fibrosis grade and NAS score) it is clear that subjects with steatosis grade 2 had higher plasma triglyceride (TG), alkylacylglycerol (DG(O)) and cholesterylester (CE) concentrations than those in whom no steatosis was detected (Fig. 1A). Plasma lipid concentrations were not associated with fibrosis grading apart from a reduction of total plasma alkyl/alkenyl-phosphatidylcholines (PC(O)) in subjects with grade 1 fibrosis compared to those without fibrosis (Fig. 1B). No significant differences in the plasma lipidome were found for NAS scores (Fig. 1C).

**Fig. 2:**
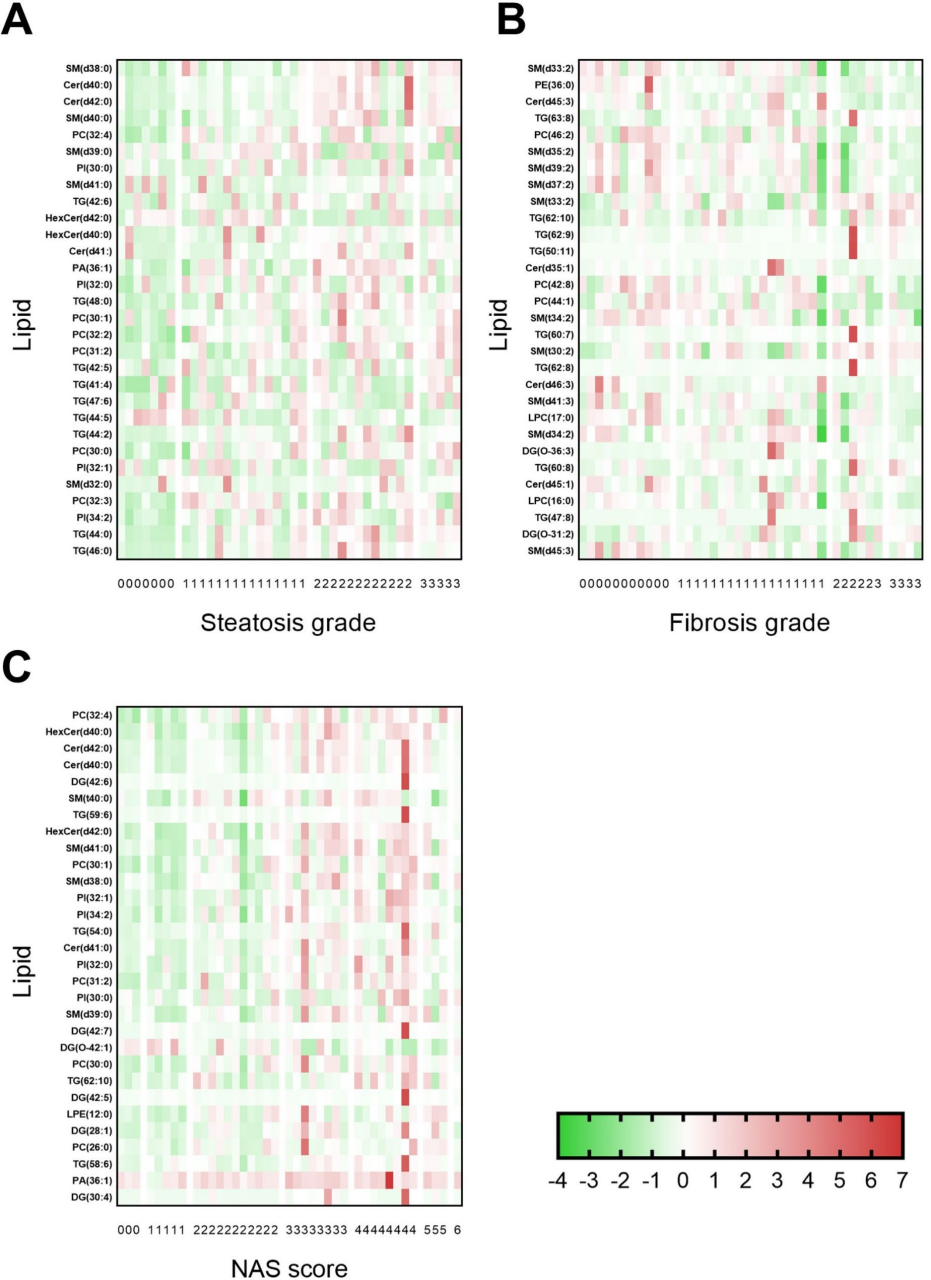
Heatmaps for the plasma concentrations for the 30 most affected individual lipids in subjects based on their (**A**) hepatic steatosis grade, (**B**) fibrosis grade, and (**C**) NAS score

With respect to the individual lipids in the lipid categories, the plasma concentrations of multiple lipids correlated with steatosis grade, fibrosis stage and NAS score (Table S3) but only a few were significant. Table 3 depicts the number of significant associated plasma lipids per lipid category showing that, for instance, 365 lipids correlated with steatosis grade. Table S44 shows the top 30 associated lipids per histology group, and the raw z-scores of these lipids are depicted in Fig. 2. After FDR correction, 126 of the 365 lipids remained correlated with steatosis grade (Table S5) of which the top 6 associated lipids were ceramides Cer(d40:0) and Cer(d42:0), sphingomyelins SM(d38:0), SM(d39:0) and SM(d40:0), and PC(32:4) (all FDR adjusted p-value of 0.0043). No plasma lipids remained correlated with fibrosis grade or NAS score.

**Table 3:** Number of lipids category that associate with steatosis grade, fibrosis grade or NAS score

		Steatosis correlated		Fibrosis correlated		NAS correlated	
**Category**	**Total (n)**	Neg. (n)	Pos. (n)	Neg. (n)	Pos. (n)	Neg. (n)	Pos. (n)
TG	366	0	153	0	8	0	40
TG(O)	97	0	17	0	0	0	11
DG	122	1	17	1	1	1	15
DG(O)	53	2	6	0	2	3	2
CE	97	0	23	0	0	0	2
PE	61	0	11	1	0	1	3
PE(O)	86	1	3	0	0	1	1
LPE	25	0	6	0	0	0	2
LPE(O)	18	0	0	0	0	0	0
PC	123	0	29	4	0	0	17
PC(O)	108	0	4	0	0	11	0
LPC	68	0	7	2	0	1	5
LPC(O)	35	1	0	0	0	2	0
PS	12	0	5	0	0	0	0
PI	31	0	10	0	0	0	15
PA	15	0	2	0	1	0	1
LPA	11	0	1	0	0	0	1
SM(d)	86	0	25	10	0	3	14
SM(t)	43	0	6	1	2	0	1
SM4(d)	26	0	1	0	0	0	0
SM4(t)	25	0	5	1	0	0	1
SPH	5	0	0	0	0	0	1
Cer	62	1	15	4	0	2	10
C1P	10	1	0	0	0	1	0
HexCer	25	0	7	0	0	0	3
Hex2Cer	15	0	5	0	0	0	3
All lipids		**7**	**358**	**24**	**14**	**26**	**148**

### Four lipids are lower in MASH

**Fig. 3:**
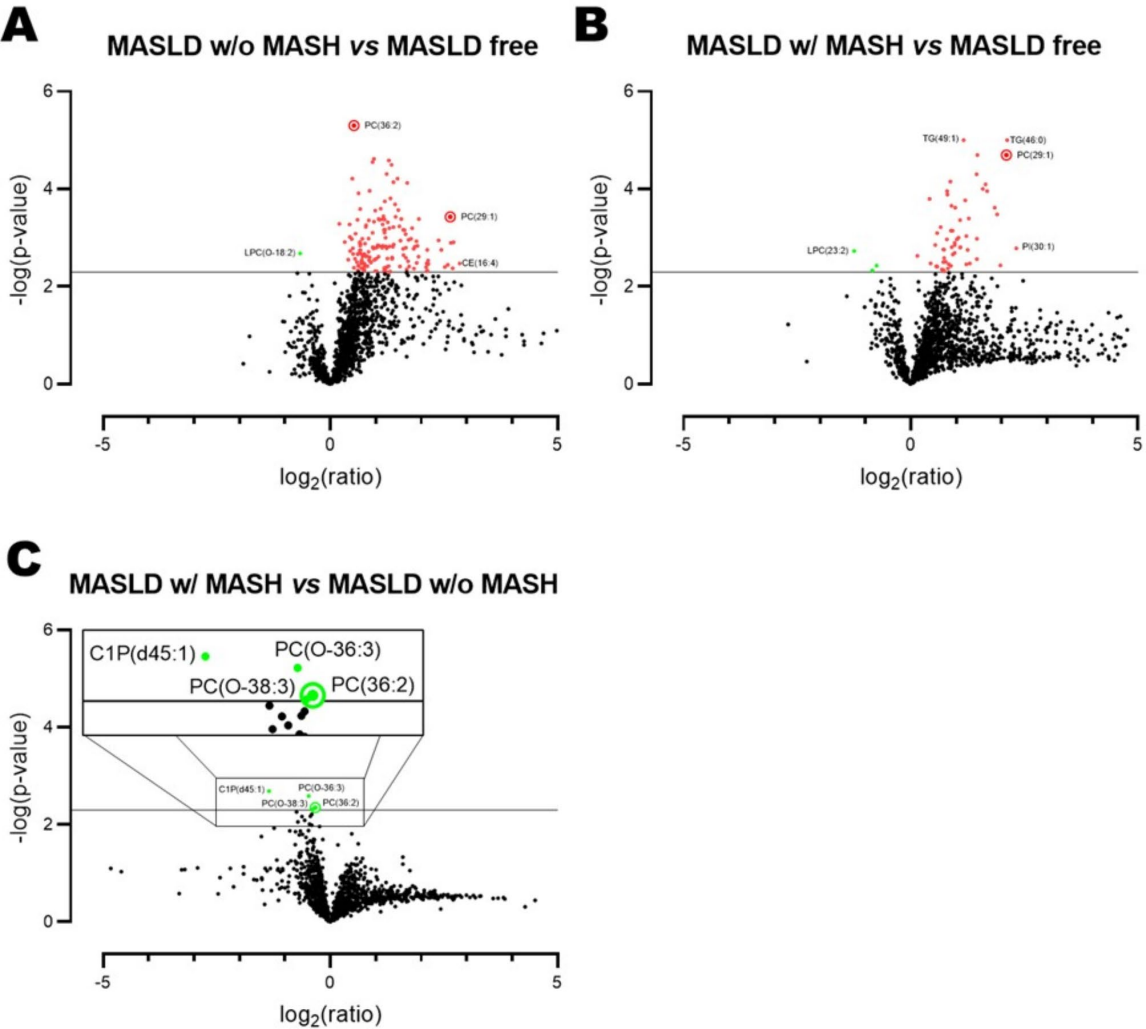
Vulcanoplots of plasma lipids when comparing (**A**) individuals with MASLD without MASH to MASLD-free subjects, (**B**) individuals with MASLD who have MASH to MASLD_free subjects, and (**C**) individuals with MASLD with MASH to those without MASH. Red dots depict lipids of which the plasma concentration is significantly higher, green dots the lipids of which the plasma concentration is significantly lower. Figure C contains a zoom-in of the four statistically significant affected plasma lipids

So far, the lipidomic analysis was done based on the histological scoring and included all 39 subjects. Next, we directly compared the plasma lipid profiles of the individuals with MASLD who have or have not progressed to MASH to those of the MASLD-free subjects (Fig. 3 and Table S6-S8). Doing so, we found that multiple PCs were elevated in the plasma of MASLD individuals without MASH compared to the MASLD-free subjects of which the most statistical relevant one was PC(36:2). In addition, the lipid that was elevated the most in MASLD individuals without MASH compared to MASLD-free subjects was CE(16:4), a highly abundant plasma lipid (Table S9).

**Fig. 4:**
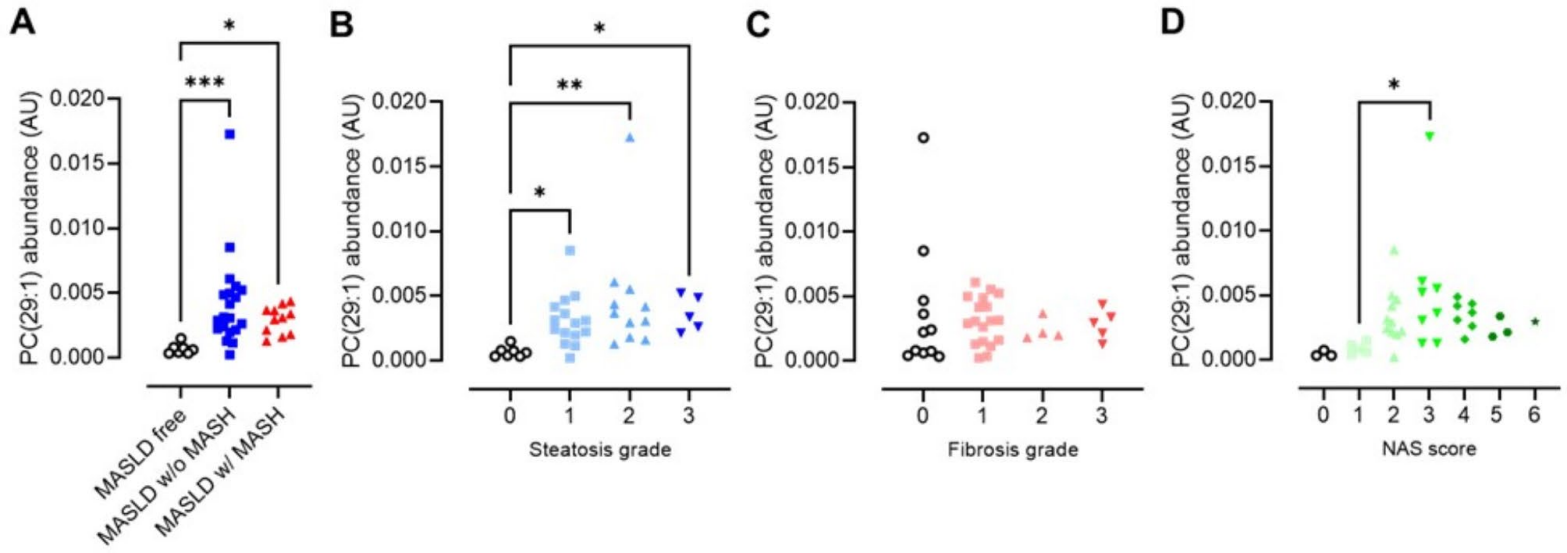
Abundance of plasma PC(29:1) in subjects with respect to their (**A**) disease state, (**B**) hepatic steatosis grade, (**C**) fibrosis grade, and (**D**) NAS score. *, *p* < 0.05; **, *p* < 0.01; ***, *p* < 0.001 (Kruskal-Wallis test)

Compared to the MASLD-free subjects, MASLD individuals who have progressed to MASH mainly had higher plasma TGs such as TG(49:1) and TG(46:0) (Fig. 3B). All plasma lipids that differed between MASLD individuals without MASH and MASLD-free subjects were also different between MASLD individuals with MASH and healthy controls. Of these, PC(29:1) was among the most increased plasma lipids in individuals with MASLD independent of MASH (Fig. 4A). Further analysis revealed that the plasma PC(29:1) concentrations depended on the steatosis and not the fibrosis grade of the liver (Fig. 4B and C). The plasma abundancy of PC(29:1) was however very low, being the 1370th of the 1625 plasma lipids detected (Table S9).

**Fig. 5:**
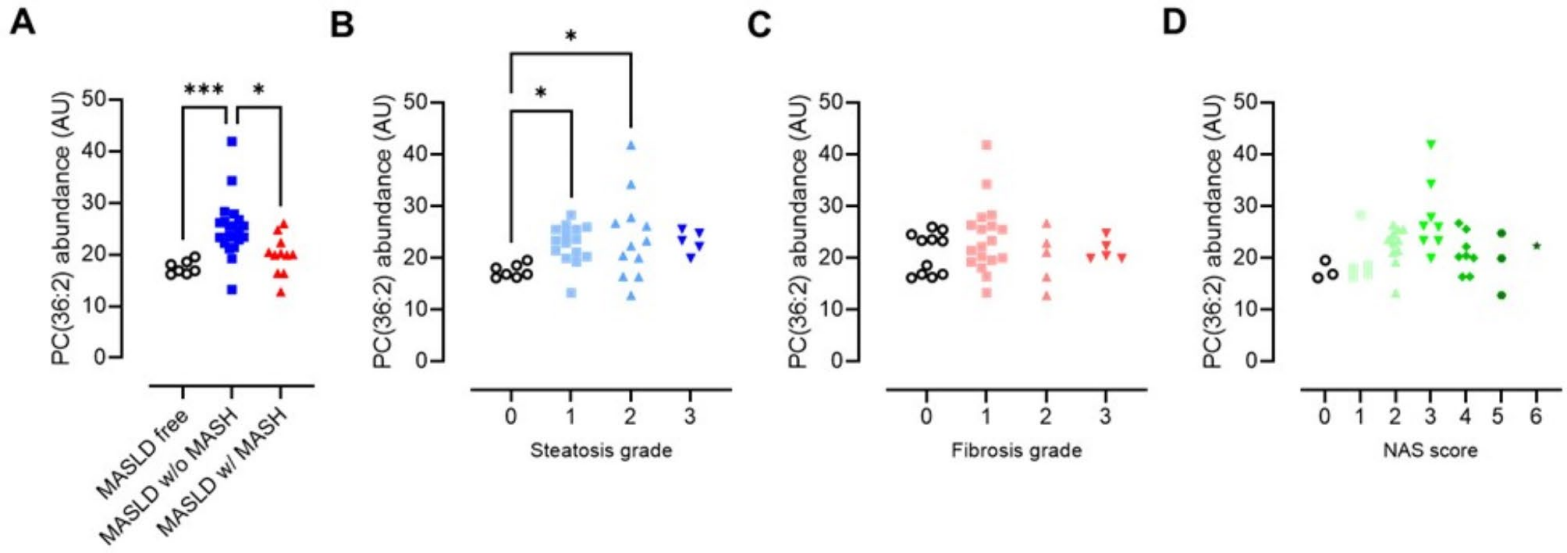
Abundance of plasma PC(36:2) in subjects with respect to their (**A**) disease state, (**B**) hepatic steatosis grade, (**C**) fibrosis grade, and (**D**) NAS score. *, *p* < 0.05; **, *p* < 0.01; ***, *p* < 0.001 (Kruskal-Wallis test)

When comparing MASLD individuals without to MASLD individuals with MASH (Fig. 3C), four plasma lipids showed a statistically significant difference in plasma concentrations, namely ceramide-1-phosphate C1P(d45:1), PC(O-36:3), PC(O-38:3) and PC(36:2). These lipids did however not remain significant after FDR correction. The plasma concentrations of all four lipids were lower in MASLD individuals with MASH than in MASLD individuals without MASH. It is however noteworthy that the plasma abundancy of the first three lipids is very low, while PC(36:2) is the 33th most abundant lipid of the 1625 detected (Table S9). In line with the notion that plasma PC(36:2) concentrations decline when MASLD progresses to MASH is the finding that this lipid in-creased with more steatosis grade but is not affected by fibrosis (Fig. 5).

### No differences in lipid saturation between subjects with or without MASLD and/or MASH

**Fig. 6:**
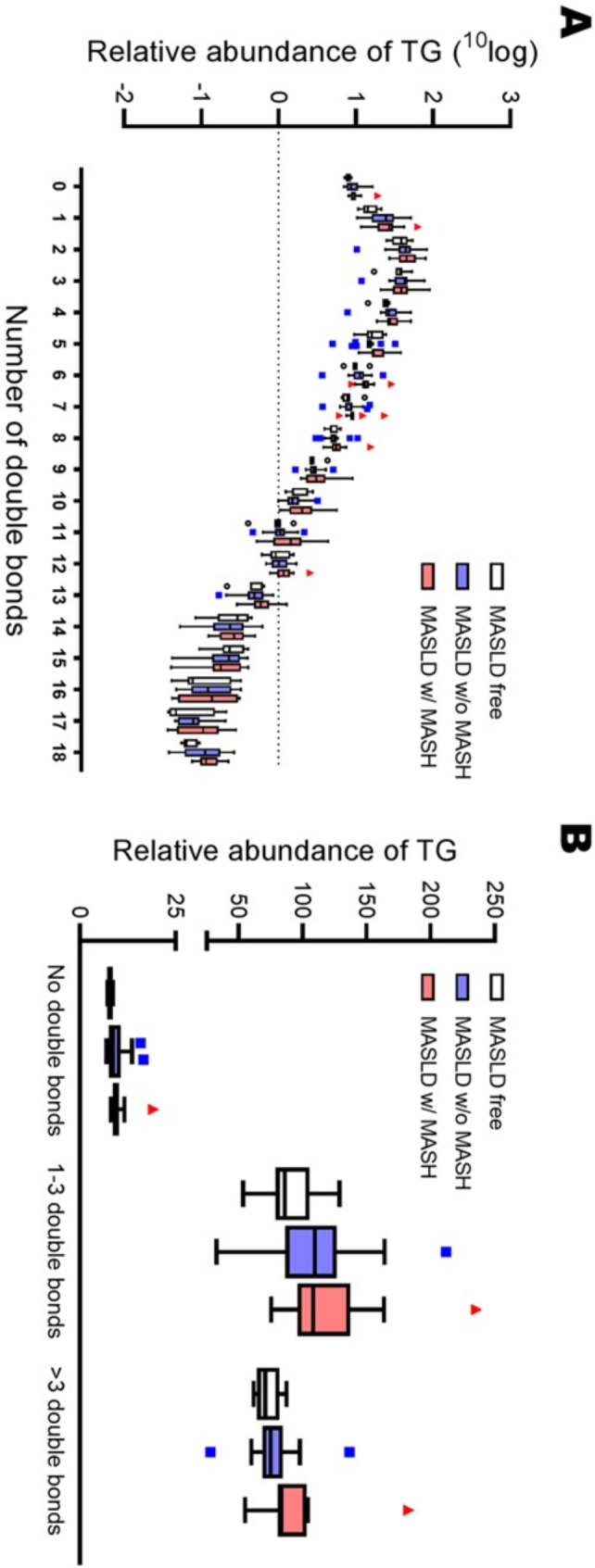
Relative abundance of plasma triglycerides based on (**A**) the number of double bonds and (**B**) grouped for either having no double bonds, up to 3 double bonds or more than 3 double bonds. Data are shown as box plots

Lipids are characterized by various chemical/physical properties, among which the saturation. As a proxy of general saturation of plasma lipids, we investigated the number of double bonds in the plasma TGs within the three groups and found no differences in saturation between MASLD-free subjects, MASLD individuals without MASH and MASLD individuals with MASH (Fig. 6).

## Discussion

MASLD is a complex multifactorial disease, in which disturbances in lipid homeostasis are key in disease onset as well as progression. Previous studies have focused on the difference in plasma lipid levels between individuals with MASLD and those without MASLD (14–20), but did not focus on differences in MASLD progression. Hence, we aimed to identify plasma lipid signatures of disease severity along the MASLD spectrum and thus performed plasma lipidomics on individuals with histologically confirmed MASLD who vary in steatosis grades, fibrosis grades and NAS scores. Moreover, we aimed to find lipid biomarkers that can distinguish individuals with MASH from those with MASLD but who have not progressed to MASH.

Our data show that differences between steatotic and non-steatotic livers is mainly reflected by elevated plasma TG, DG(O), and CE concentrations. This is likely caused by elevated VLDL secretion from steatotic livers, as all three lipid classes are well known components of the hydrophobic core of VLDL. The fact that lipoprotein surface lipids such as phospholipids do not differ suggest that the VLDL particles were enlarged. Secretion of large VLDL particles has been observed before in humans with elevated liver fat as well as those with type 2 diabetes [28]. The aggregation of adipose tissue derived fatty acids in MASLD is often followed by the upregulated incorporation of fatty acids into TGs in lipid droplets, which might result in autophagy of these lipid droplets. Both processes are associated with elevated VLDL secretion [13, 29].

Detailed analyses showed that plasma concentrations of ceramides and sphingomyelins were predominantly affected by the steatosis grade. The increased plasma ceramide concentration in subjects with steatotic livers is in line with previous studies [30, 31]. Sphingomyelins and ceramides are classes of sphingolipids, bioactive lipids that are found ubiquitously and play an important role in, amongst others, cell growth and survival, and immune responses [32]. Ceramides are the central molecules in sphingolipid metabolism and have previously been shown to be linked to metabolic disturbances such as IR, oxidative stress, and inflammation [33–36]. They disrupt insulin sensitivity and mitochondrial metabolism, which leads to metabolic derangement and cell death [37, 38]. Sphingolipid metabolites have previously been associated with key processes in MASLD pathophysiology, such as the initiation of proinflammatory events leading to fibrosis and necroinflammation [39]. The association between high ceramide levels and increased steatosis grade has also been confirmed in vivo, as ceramide inhibitors attenuated MASLD in rats fed a high fat diet [40]. Altogether, the present study underscores the crucial roles of sphingolipids in MASLD.

The lipids that correlated with steatosis grades and NAS scores were predominantly saturated ones. More specifically, 16 out of the top 30 lipids that correlated with steatosis were monounsaturated. This is in line with previous studies reporting increased plasma concentrations of saturated and monounsaturated lipids in individuals with hepatic steatosis [20, 41–43]. In our study, this was however not reflected by differences in the number of double bonds in TGs in the plasma of MASLD subjects compared to healthy controls. This is likely due to the heterogeneity of lipid species within the TG lipid class.

PC(29:1) might be a candidate lipid biomarker for MASLD, since the plasma concentration of this lipid was higher in individuals with MASLD than in those without MASLD. Of interest, our data suggest that steatosis drives the elevated PC(29:1) concentration. This specific lipid species has not previously been earmarked as a potential MASLD biomarker which might be due to the its very low plasma abundance. Future studies are therefore needed to explore whether this rare lipid can indeed serve as a biomarker or whether its presence in the current study is just a spurious finding.

To discriminate between MASLD subjects with and without MASH, four lipids can be considered, namely C1P(d45:1), PC(O-36:3), PC(O-38:3) and PC(36:2) which all have lower plasma concentrations in those with MASH than in those without MASH, albeit that they did not remain significant after FDR correction. Of these lipids, the first three ones are very rare ones making them not easily applicable as biomarkers. PC(36:2), in contrast, is a common plasma lipid of which the plasma concentrations have for instance been reported to be higher in MASLD individuals with hypertension compared to those without hypertension [31]. A couple of studies have reported PC(36:2) molecules to at least associate with features corresponding to MASLD and/or MASH. For instance, PC(18:0/18:2) is a major component of HDL particles [44] whose concentrations are normally lower in subjects with MASLD albeit that this not the case in our cohort. Moreover, PC(18:1/18:1) has been shown to lower in a progressed hepatocellular carcinoma (HCC) mouse model [45]. Altogether, these data underscore the potential of plasma PC(36:2) concentrations to serve as a MASH proxy or biomarker. Successful implementation of this lipid as a biomarker is however hampered by the fact that it cannot be measured routinely since high throughput assay are not available. Hence, a laboratory depends on specialized mass spectrometry (MS) techniques that are labor intensive, expensive and not accessible to all.

The present study comes with some limitations of which the most important one is the limited sample size. This is mainly due to the fact that MASLD, although rather prevalent, is not routinely diagnosed. This might be caused by the lack of awareness of MASLD in [46] but also by the fact that reliable non-invasive liver tests are lacking, limiting the inclusion of MASLD subjects. Another limitation is the imbalance in the distribution of subjects over the groups. While the MASLD-free subjects were mainly females with an average age of 36, the MASLD subjects with and without MASH were males with an average age of 55 and 52 years, respectively. The skewed distribution of the sexes over the groups might have influenced the lipidomics results. For instance, we found that plasma concentrations of ceramides and sphingomyelins are higher in subjects with steatosis, which might be attributable to the relatively overrepresentation of men since they have relatively higher plasma ceramide and sphingomyelin concentrations [47]. Age might be another confounder. Almeida et al. [48] reviewed the evidence of specific plasma lipids as biomarkers of physiological aging (and not pathophysiological ageing due to age-related diseases) and they conclude that elevated plasma concentrations of PC(O-32:1) [49], elevated plasma SM(24:1), SM(16:0) and reduced LPC(18:2) and LPC(20:4) [50], and reduced sphingolipids and elevated sterols as well as glycerolipids [51] might all be possible biomarkers of healthy ageing. However, in our study only LPC(18:2) and LPC(20:4) were detected at relative high abundancy in the plasma and none of the suggested age-dependent lipids did differ between the three different groups of subjects. Hence, these results strongly suggest that age was not a significant confounder in our lipidomic analysis. Altogether, replication of the lipidomics profiling in larger, well-defined MASLD cohorts with a properly balanced distribution of sex and age is urgently awaited in order to strengthen the result from the present study.

## Conclusions

We investigated the association between plasma lipid profiles and histologically characterized MASLD disease severity. Our results elucidate the association between hepatic steatosis and specific lipid species such as sphingolipids. PC(29:1) may have potential as a biomarker for MASLD and PC(36:2) may have the potential discriminate MASH from isolated steatosis. However, the lack of throughput assays hamper implementation of specific lipids as biomarkers. Our cohort was limited in size, hence replication is needed in larger, well-defined MASLD cohorts.

## Electronic supplementary material

Below is the link to the electronic supplementary material.

Supplementary Material 1

## Data Availability

No datasets were generated or analysed during the current study.

## Abbreviations

ALT: Alanine Aminotransferase
AST: Aspartate Aminotransferase
BMI: Body Mass Index
BMP: Bis(monoacylglycero)phosphate
C1P: Ceramide-1-Phosphate
CE: Cholesterylester
Cer: Ceramide
CRN: Clinical Research Network
DG: Diacylglycerol
DG(O): Alkylacylglycerol
FAO: Fatty Acid Oxidation
GGT: Gamma-Glutamyl Transferase
HbA1c: Hemoglobin-A1c
HCC: Hepatocellular Carcinoma
HDL-c: High Density Lipoprotein cholesterol
IR: Insulin Resistance
LDL-c: Low Density Lipoprotein Cholesterol
LPC: Lysophoshatidylcholine
MASH: Metabolic Dysfunction-Associated Steatohepatitis
MASLD: Metabolic Dysfunction-Associated Steatotic Liver Disease
MS: Mass Spectrometer
NAFLD: Non-Alcoholic Fatty Liver Disease
NAS: NAFLD Activity Score
PC(O): alkyl/alkenyl-phosphatidylcholines
SM: Sphingomyelin
T2DM: Type 2 Diabetes Mellitus
TC: Total Cholesterol
TG: Triacylglycerol
VLDL: Very Low Density Lipoprotein
