# Supplementary material for: The plasma lipidome varies with the severity of metabolic dysfunction-associated steatotic liver disease
Source: Lipids Health Dis. 2024 Dec 18;23:402. doi: 10.1186/s12944-024-02380-x (PMC11653580; doi:10.1186/s12944-024-02380-x)
Supplement: Supplementary file 1 — Supplementary Material 1 [file 12944_2024_2380_MOESM1_ESM.docx]

Supplemental data for:

**The plasma lipidome varies with the severity of metabolic dysfunction-associated steatotic liver disease**

Heymann et al.

**Table S1.** Profiles of the 39 subjects.

| Group | Subject | Institute | Sex | Age  (years) | Steatosis  (%) | Grading | | | | | |
| --- | --- | --- | --- | --- | --- | --- | --- | --- | --- | --- | --- |
|  |  |  |  |  |  | Steatosis | Fibrosis | NAS | Inflamm. | Ball. |  |
| MASLD-free | 1 | MUMC+ | Female | 55 | 0 | 0 | 0 | 1 | 1 | 0 |  |
|  | 2 | MUMC+ | Female | 28 | 0 | 0 | 0 | 1 | 1 | 0 |  |
|  | 3 | MUMC+ | Female | 30 | 0 | 0 | 0 | 0 | 0 | 0 |  |
|  | 4 | MUMC+ | Female | 29 | 0 | 0 | 1 | 1 | 1 | 0 |  |
|  | 5 | MUMC+ | Female | 45 | 0 | 0 | 1 | 0 | 0 | 0 |  |
|  | 6 | MUMC+ | Male | 58 | 0 | 0 | 0 | 1 | 1 | 0 |  |
|  | 7 | MUMC+ | Female | 36 | 0 | 0 | 0 | 0 | 0 | 0 |  |
| MASLD without MASH | 1 | AUMC | Male | 55 | 10 | 1 | 0 | 2 | 1 | 0 |  |
|  | 2 | AUMC | Male | 61 | 50 | 2 | 1 | 3 | 1 | 0 |  |
|  | 3 | AUMC | Male | 48 | 15 | 1 | 0 | 2 | 1 | 0 |  |
|  | 4 | AUMC | Male | 61 | 20 | 1 | 1 | 2 | 1 | 0 |  |
|  | 5 | AUMC | Female | 40 | 55 | 2 | 2 | 4 | 2 | 0 |  |
|  | 6 | AUMC | Male | 51 | 30 | 1 | 1 | 1 | 0 | 0 |  |
|  | 7 | AUMC | Male | 53 | 60 | 2 | 0 | 3 | 1 | 0 |  |
|  | 8 | AUMC | Female | 54 | 20 | 1 | 1 | 2 | 1 | 0 |  |
|  | 9 | AUMC | Male | 36 | 20 | 1 | 1 | 2 | 1 | 0 |  |
|  | 10 | MUMC | Male | 56 | 20 | 1 | 1 | 2 | 1 | 0 |  |
|  | 11 | MUMC | Male | 21 | 80 | 3 | 1 | 4 | 1 | 0 |  |
|  | 12 | AUMC | Male | 56 | 40 | 2 | 1 | 3 | 1 | 0 |  |
|  | 13 | AUMC | Male | 45 | 55 | 2 | 1 | 3 | 1 | 0 |  |
|  | 14 | AUMC | Female | 63 | 35 | 2 | 1 | 3 | 1 | 0 |  |
|  | 15 | MUMC+ | Male | 53 | 90 | 3 | 1 | 3 | 1 | 0 |  |
|  | 16 | AUMC | Male | 58 | 66 | 3 | 1 | 4 | 1 | 0 |  |
|  | 17 | AUMC | Male | 56 | 10 | 1 | 1 | 2 | 1 | 0 |  |
|  | 18 | AUMC | Male | 59 | 25 | 1 | 2 | 2 | 1 | 0 |  |
|  | 19 | AUMC | Male | 55 | 25 | 1 | 2 | 2 | 1 | 0 |  |
|  | 20 | MUMC+ | Female | 65 | 5 | 1 | 0 | 2 | 1 | 0 |  |
|  | 21 | AUMC | Male | 34 | 10 | 1 | 0 | 2 | 1 | 0 |  |
| MASLD with MASH | 1 | AUMC | Male | 57 | 15 | 1 | 0 | 3 | 1 | 1 |  |
|  | 2 | AUMC | Female | 50 | 60 | 2 | 1 | 4 | 1 | 1 |  |
|  | 3 | AUMC | Male | 50 | 25 | 1 | 3 | 3 | 1 | 1 |  |
|  | 4 | AUMC | Male | 64 | 35 | 2 | 3 | 4 | 1 | 1 |  |
|  | 5 | MUMC+ | Female | 52 | 70 | 3 | 3 | 5 | 1 | 1 |  |
|  | 6 | MUMC+ | Male | 27 | 60 | 2 | 1 | 4 | 1 | 1 |  |
|  | 7 | MUMC+ | Male | 54 | 40 | 2 | 3 | 6 | 2 | 2 |  |
|  | 8 | MUMC+ | Male | 53 | 15 | 1 | 1 | 4 | 2 | 1 |  |
|  | 9 | MUMC+ | Male | 35 | ? | 2 | 2 | 5 | 1 | 2 |  |
|  | 10 | MUMC+ | Female | 60 | 70 | 3 | 3 | 5 | 1 | 1 |  |
|  | 11 | AUMC | Male | 35 | 60 | 2 | 2 | 4 | 1 | 1 |  |

**Table S2.** Overview of the lipids and their categories in the database

| Category | Subcategory | Short | No. of lipids |
| --- | --- | --- | --- |
| Glycerolipids | Triacylglycerols | TG | 366 |
|  | Alkyldiacylglycerols | TG(O) | 97 |
|  | Diacylglycerols | DG | 122 |
|  | Alkylacylglycerols | DG(O) | 53 |
| Cholesterylesters | Cholesterylesters | CE | 97 |
| Glycerophospholipids | Phosphatidylethanolamines | PE | 61 |
|  | Alkyl/alkenyl-phosphatidylethanolamines | PE(O) | 86 |
|  | Lysophosphatidylethanolamines | LPE | 25 |
|  | Alkyl/alkenyl-lysophosphatidylethanolamines | LPE(O) | 18 |
|  | Phosphatidylcholines | PC | 123 |
|  | Alkyl/alkenyl-phosphatidylcholines | PC(O) | 108 |
|  | Lysophosphatidylcholines | LPC | 68 |
|  | Alkyl/alkenyl-lysophosphatidylcholines | LPC(O) | 35 |
|  | Phosphatidylserines | PS | 12 |
|  | Phosphatidylinositols | PI | 31 |
|  | Phosphatidic acids | PA | 15 |
|  | Lysophosphatic acids | LPA | 11 |
| Sphingolipids | Sphingomyelins | SM(d) | 86 |
|  | Hydroxysphingomyelins | SM(t) | 43 |
|  | Sulfatides | SM4(d) | 26 |
|  | Hydroxysulfatides | SM4(t) | 25 |
|  | Sphingoids | SPH | 5 |
|  | Ceramides | Cer | 62 |
|  | Ceramide-1-phosphates | C1P | 10 |
|  | Hexosylceramides | HexCer | 25 |
|  | Dihexosylceramides | Hex2Cer | 15 |

**Table S3.** Number of lipids per lipid category that associate with steatosis grade, fibrosis grade or NAS score.

|  | | Steatosis correlated (n, %) | | Fibrosis correlated (n,%) | | NAS correlated (n, %) | |
| --- | --- | --- | --- | --- | --- | --- | --- |
| **Lipid category** | No. lipids | Negative | Positive | Negative | Positive | Negative | Positive |
| TG | 366 | 27, 7 | 339, 93 | 58, 16 | 308, 84 | 37, 10 | 329, 90 |
| TG(O) | 97 | 12, 12 | 85, 88 | 35, 36 | 62, 64 | 14, 14 | 63, 86 |
| DG | 122 | 16, 13 | 106, 87 | 75, 61 | 47, 39 | 13, 11 | 109, 89 |
| DG(O) | 53 | 19, 36 | 34, 64 | 32, 60 | 21, 40 | 24, 45 | 29, 55 |
| CE | 97 | 1, 1 | 96, 99 | 83, 86 | 14, 14 | 5, 5 | 92, 95 |
| PE | 61 | 2, 3 | 59, 97 | 45, 74 | 20, 26 | 5, 8 | 56, 92 |
| PE(O) | 86 | 16, 19 | 70, 81 | 51, 59 | 35, 41 | 16, 19 | 70, 81 |
| LPE | 25 | 3, 12 | 22, 88 | 15, 60 | 10, 40 | 3, 12 | 22, 88 |
| LPE(O) | 18 | 13, 72 | 5, 28 | 17, 94 | 1, 6 | 9, 50 | 9, 50 |
| PC | 123 | 6, 5 | 117, 95 | 77, 63 | 46, 37 | 24, 20 | 99, 80 |
| PC(O) | 108 | 49, 45 | 59, 55 | 77, 71 | 31, 29 | 69, 64 | 39, 36 |
| LPC | 68 | 18, 26 | 50, 74 | 40, 59 | 28, 41 | 23, 34 | 45, 66 |
| LPC(O) | 35 | 20, 57 | 15, 43 | 25, 71 | 10, 29 | 23, 66 | 12, 34 |
| PS | 12 | 1, 8 | 11, 92 | 8, 67 | 4, 33 | 2, 17 | 10, 83 |
| PI | 31 | 0, 0 | 31, 100 | 19, 61 | 12, 39 | 1, 3 | 30, 97 |
| PA | 15 | 0, 0 | 15, 100 | 4, 27 | 11, 73 | 3, 20 | 12, 80 |
| LPA | 11 | 1, 9 | 10 , 91 | 1, 9 | 10, 91 | 1, 9 | 10, 91 |
| SM(d) | 86 | 23, 27 | 63, 73 | 53, 62 | 33, 38 | 39, 45 | 47, 55 |
| SM(t) | 43 | 7, 16 | 36, 84 | 32, 74 | 11, 26 | 18, 42 | 25, 58 |
| SM4(d) | 26 | 6, 23 | 20, 77 | 23, 88 | 3,12 | 9, 35 | 17, 65 |
| SM4(t) | 25 | 1, 4 | 24, 96 | 24, 96 | 1, 4 | 1, 4 | 24, 96 |
| SPH | 5 | 1, 20 | 4, 80 | 3, 60 | 2, 40 | 3, 60 | 2, 40 |
| Cer | 62 | 16, 26 | 46, 74 | 47, 76 | 15, 24 | 26, 42 | 36, 58 |
| C1P | 10 | 7, 70 | 3, 30 | 6, 60 | 4, 40 | 7, 70 | 3, 30 |
| HexCer | 25 | 4, 16 | 21, 84 | 25, 100 | 0, 0 | 4, 16 | 21, 84 |
| Hex2Cer | 15 | 3, 20 | 12, 80 | 8, 53 | 7, 47 | 7, 47 | 8, 53 |

**Table S4.** Top 30 lipids significantly associated with steatosis grade, fibrosis grade or NAS score

| Steatosis grade | | | | Fibrosis grade | | | | | NAS score | | | | | |
| --- | --- | --- | --- | --- | --- | --- | --- | --- | --- | --- | --- | --- | --- | --- |
| Lipid | Corr. Coeff. | -log(p) |  | Lipid | Corr. Coeff. | -log(p) |  | Lipid | | Corr. Coeff. | | -log(p) |  |  |
| **Sphingomyelins** | | |  | **Sphingomyelins** | | |  | | **Phosphatidylcholines** | | | | | |
| SM(d38:0) | 0.66 | 5.27 |  | SM(d33:2) | -0.51 | 3.06 |  | PC(32:4) | | 0.57 | | 3.75 |  |  |
| SM(d40:0) | 0.64 | 4.86 |  | SM(d35:2) | -0.39 | 1.84 |  | PC(30:1) | | 0.51 | | 3.05 |  |  |
| SM(d39:0) | 0.63 | 4.79 |  | SM(d39:2) | -0.39 | 1.83 |  | PC(31:2) | | 0.48 | | 2.75 |  |  |
| SM(d41:0) | 0.62 | 4.57 |  | SM(d37:2) | -0.38 | 1.76 |  | PC(30:0) | | 0.46 | | 2.47 |  |  |
| SM(d32:0) | 0.55 | 3.51 |  | SM(d41:3) | -0.34 | 1.46 |  | PC(26:0) | | 0.44 | | 2.30 |  |  |
| **Ceramides** | | |  | SM(d34:2) | -0.34 | 1.45 |  | **Hexosylceramides** | | | | | |  |
| Cer(d40:0) | 0.64 | 4.94 |  | SM(d45:3) | -0.33 | 1.36 |  | HexCer(d40:0) | | 0.55 | | 3.59 |  |  |
| Cer(d42:0) | 0.64 | 4.87 |  | **Hydroxysphingomyelins** | | |  | | HexCer(d42:0) | | 0.52 | 3.15 |  |  |
| Cer(d41:0) | 0.59 | 4.10 |  | SM(t33:2) | 0.37 | 1.71 |  | **Ceramides** | | | | | |  |
| **Hexosylceramides** | | |  | SM(t34:2) | -0.35 | 1.56 |  | Cer(d42:0) | | 0.55 | | 3.57 |  |  |
| HexCer(d42:0) | 0.61 | 4.36 |  | SM(t30:2) | 0.35 | 1.52 |  | Cer(d40:0) | | 0.54 | | 3.46 |  |  |
| HexCer(d40:0) | 0.59 | 4.18 |  | **Phosphatidylethanolamines** | | |  | | Cer(d41:0) | | 0.49 | 2.80 |  |  |
| **Phosphatidylcholines** | | |  | PE(36:0) | -0.5 | 2.93 |  | **Diacylglycerols** | | | | | |  |
| PC(32:4) | 0.64 | 4.85 |  | **Ceramides** | | |  | | DG(42:6) | | 0.54 | 3.40 |  |  |
| PC(30:1) | 0.57 | 3.82 |  | Cer(d45:3) | -0.44 | 2.31 |  | DG(42:7) | | 0.47 | | 2.59 |  |  |
| PC(32:2) | 0.57 | 3.80 |  | Cer(d35:1) | -0.37 | 1.65 |  | DG(42:5) | | 0.45 | | 2.40 |  |  |
| PC(31:2) | 0.56 | 3.66 |  | Cer(d46:3) | -0.34 | 1.48 |  | DG(28:1) | | 0.44 | | 2.31 |  |  |
| PC(30:0) | 0.55 | 3.53 |  | Cer(d45:1) | -0.33 | 1.39 |  | DG(30:4) | | 0.44 | | 2.26 |  |  |
| PC(32:3) | 0.55 | 3.51 |  | **Triacylglycerols** | | |  | | **Sphingomyelins** | | | | | |
| **Phosphatidylinositols** | | |  | TG(63:8) | 0.43 | 2.23 |  | SM(d40:0) | | 0.54 | | 3.40 |  |  |
| PI(30:0) | 0.62 | 4.64 |  | TG(62:10) | 0.37 | 1.71 |  | SM(d41:0) | | 0.51 | | 3.08 |  |  |
| PI(32:0) | 0.58 | 3.93 |  | TG(62:9) | 0.37 | 1.69 |  | SM(d38:0) | | 0.51 | | 3.01 |  |  |
| PI(32:1) | 0.55 | 3.52 |  | TG(50:11) | 0.37 | 1.67 |  | SM(d39:0) | | 0.48 | | 2.68 |  |  |
| PI(34:2) | 0.54 | 3.45 |  | TG(60:7) | 0.35 | 1.54 |  | **Triacylglycerols** | | | | | |  |
| **Triacylglycerols** | | |  | TG(62:8) | 0.34 | 1.49 |  | TG(59:6) | | 0.52 | | 3.19 |  |  |
| TG(42:6) | 0.61 | 4.40 |  | TG(60:8) | 0.33 | 1.41 |  | TG(54:0) | | 0.49 | | 2.86 |  |  |
| TG(48:0) | 0.57 | 3.88 |  | TG(47:8) | 0.33 | 1.38 |  | TG(62:10) | | 0.46 | | 2.46 |  |  |
| TG(42:5) | 0.56 | 3.65 |  | **Phosphatidylcholines** | | |  | | TG(58:6) | | 0.44 | 2.28 |  |  |
| TG(41:4) | 0.56 | 3.61 |  | PC(46:2) | -0.41 | 1.98 |  | **Phosphatidylinositols** | | | | | |  |
| TG(47:6) | 0.56 | 3.61 |  | PC(42:8) | -0.36 | 1.62 |  | PI(32:1) | | 0.51 | | 2.99 |  |  |
| TG(44:5) | 0.55 | 3.57 |  | PC(44:1) | -0.36 | 1.59 |  | PI(34:2) | | 0.50 | | 2.95 |  |  |
| TG(44:2) | 0.55 | 3.54 |  | **Lysophosphatidylcholines** | | |  | | PI(32:0) | | 0.49 | 2.80 |  |  |
| TG(44:0) | 0.54 | 3.44 |  | LPC(17:0) | -0.34 | 1.45 |  | PI(30:0) | | 0.48 | | 2.75 |  |  |
| TG(46:0) | 0.54 | 3.44 |  | LPC(16:0) | -0.33 | 1.38 |  | **Alkylacylglycerols** | | | | | |  |
| **Phosphatidic acids** | | |  | **Alkylacylglycerols** | | |  | | DG(O-42:1) | | -0.46 | 2.50 |  |  |
| PA(36:1) | 0.58 | 3.96 |  | DG(O-36:3) | 0.33 | 1.42 |  | **Lysophosphatidylethanolamines** | | | | | |  |
|  | | |  | DG(O-31:2) | 0.33 | 1.37 |  | LPE(12:0) | | 0.44 | | 2.32 |  |  |
|  |  |  |  |  | | |  | | **Phosphatidic acids** | | | | | |
|  |  |  |  |  |  |  |  |  | PA(36:1) | | 0.44 | 2.26 |  |  |

Table S5. All 126 lipids that associated with steatosis grade after FDR correction

| Lipid | Corr. Coeff. | p-value | FDR adjusted p-value |
| --- | --- | --- | --- |
| **Ceramides** | | | |
| Cer(d40:0) | 0.6402 | 0.0000 | 0.0043 |
| Cer(d42:0) | 0.6363 | 0.0000 | 0.0043 |
| Cer(d41:0) | 0.5889 | 0.0001 | 0.0108 |
| Cer(d38:0) | 0.4951 | 0.0014 | 0.0285 |
| Cer(d39:0) | 0.5285 | 0.0005 | 0.0224 |
| Cer(d40:1) | 0.4659 | 0.0028 | 0.0407 |
| **Phosphatidylcholines** | | | |
| PC(32:4) | 0.6351 | 0.0000 | 0.0043 |
| PC(30:1) | 0.5701 | 0.0002 | 0.0152 |
| PC(32:2) | 0.5686 | 0.0002 | 0.0152 |
| PC(31:2) | 0.5584 | 0.0002 | 0.0188 |
| PC(30:0) | 0.5488 | 0.0003 | 0.0188 |
| PC(32:3) | 0.5472 | 0.0003 | 0.0188 |
| PC(26:0) | 0.4981 | 0.0013 | 0.0285 |
| PC(38:3) | 0.4823 | 0.0019 | 0.0340 |
| PC(29:0) | 0.4789 | 0.0020 | 0.0349 |
| PC(29:1) | 0.5235 | 0.0006 | 0.0233 |
| PC(28:1) | 0.5162 | 0.0008 | 0.0240 |
| PC(28:2) | 0.5102 | 0.0009 | 0.0263 |
| PC(39:3) | 0.5016 | 0.0011 | 0.0283 |
| **Sphingomyelins** | | | |
| SM(d38:0) | 0.6577 | 0.0000 | 0.0043 |
| SM(d39:0) | 0.6317 | 0.0000 | 0.0043 |
| SM(d40:0) | 0.6355 | 0.0000 | 0.0043 |
| SM(d41:0) | 0.6186 | 0.0000 | 0.0055 |
| SM(d32:0) | 0.5474 | 0.0003 | 0.0188 |
| SM(d43:0) | 0.5310 | 0.0005 | 0.0216 |
| SM(d40:1) | 0.4896 | 0.0016 | 0.0301 |
| SM(d30:0) | 0.4809 | 0.0019 | 0.0344 |
| SM(d44:0) | 0.4646 | 0.0029 | 0.0411 |
| SM(d26:1) | 0.4623 | 0.0030 | 0.0423 |
| SM(d28:2) | 0.4554 | 0.0036 | 0.0465 |
| **Phosphatidylinositols** | | | |
| PI(30:0) | 0.6226 | 0.0000 | 0.0053 |
| PI(32:0) | 0.5779 | 0.0001 | 0.0136 |
| PI(32:1) | 0.5485 | 0.0003 | 0.0188 |
| PI(34:2) | 0.5433 | 0.0004 | 0.0192 |
| PI(30:1) | 0.4703 | 0.0025 | 0.0382 |
| PI(38:6) | 0.4997 | 0.0012 | 0.0283 |
| PI(31:0) | 0.4565 | 0.0035 | 0.0461 |
| **Hexosylceramides** | | | |
| HexCer(d42:0) | 0.6056 | 0.0000 | 0.0072 |
| HexCer(d40:0) | 0.5946 | 0.0001 | 0.0098 |
| **Triacylglycerols** | | | |
| TG(42:6) | 0.6082 | 0.0000 | 0.0072 |
| TG(48:0) | 0.5743 | 0.0001 | 0.0143 |
| TG(42:5) | 0.5576 | 0.0002 | 0.0188 |
| TG(41:4) | 0.5551 | 0.0002 | 0.0188 |
| TG(47:6) | 0.5550 | 0.0002 | 0.0188 |
| TG(44:5) | 0.5524 | 0.0003 | 0.0188 |
| TG(44:2) | 0.5500 | 0.0003 | 0.0188 |
| TG(44:0) | 0.5424 | 0.0004 | 0.0192 |
| TG(46:0) | 0.5419 | 0.0004 | 0.0192 |
| TG(38:2) | 0.5403 | 0.0004 | 0.0192 |
| TG(43:5) | 0.5402 | 0.0004 | 0.0192 |
| TG(46:6) | 0.5398 | 0.0004 | 0.0192 |
| TG(44:3) | 0.5346 | 0.0005 | 0.0209 |
| TG(42:1) | 0.5344 | 0.0005 | 0.0209 |
| TG(45:6) | 0.4968 | 0.0013 | 0.0285 |
| TG(43:4) | 0.4961 | 0.0013 | 0.0285 |
| TG(38:3) | 0.4959 | 0.0013 | 0.0285 |
| TG(47:8) | 0.4959 | 0.0013 | 0.0285 |
| TG(40:4) | 0.4948 | 0.0014 | 0.0285 |
| TG(52:0) | 0.4945 | 0.0014 | 0.0285 |
| TG(47:4) | 0.4942 | 0.0014 | 0.0285 |
| TG(48:6) | 0.4932 | 0.0014 | 0.0289 |
| TG(47:2) | 0.4920 | 0.0015 | 0.0291 |
| TG(48:5) | 0.4912 | 0.0015 | 0.0293 |
| TG(42:4) | 0.4873 | 0.0017 | 0.0316 |
| TG(47:3) | 0.4852 | 0.0017 | 0.0326 |
| TG(51:1) | 0.4824 | 0.0019 | 0.0340 |
| TG(48:3) | 0.4821 | 0.0019 | 0.0340 |
| TG(47:1) | 0.4797 | 0.0020 | 0.0349 |
| TG(50:7) | 0.4765 | 0.0022 | 0.0361 |
| TG(48:4) | 0.4764 | 0.0022 | 0.0361 |
| TG(38:1) | 0.4722 | 0.0024 | 0.0378 |
| TG(40:1) | 0.4716 | 0.0024 | 0.0378 |
| TG(43:6) | 0.4736 | 0.0023 | 0.0378 |
| TG(49:6) | 0.4730 | 0.0024 | 0.0378 |
| TG(56:1) | 0.4719 | 0.0024 | 0.0378 |
| TG(45:1) | 0.4715 | 0.0024 | 0.0378 |
| TG(50:1) | 0.4715 | 0.0024 | 0.0378 |
| TG(50:0) | 0.4712 | 0.0025 | 0.0378 |
| TG(44:1) | 0.5340 | 0.0005 | 0.0209 |
| TG(45:2) | 0.5330 | 0.0005 | 0.0209 |
| TG(45:4) | 0.5278 | 0.0006 | 0.0224 |
| TG(46:7) | 0.5272 | 0.0006 | 0.0224 |
| TG(45:3) | 0.5234 | 0.0006 | 0.0233 |
| TG(48:1) | 0.5221 | 0.0007 | 0.0233 |
| TG(42:2) | 0.5220 | 0.0007 | 0.0233 |
| TG(43:2) | 0.5217 | 0.0007 | 0.0233 |
| TG(43:3) | 0.5199 | 0.0007 | 0.0240 |
| TG(44:6) | 0.5191 | 0.0007 | 0.0240 |
| TG(54:0) | 0.5178 | 0.0007 | 0.0240 |
| TG(46:4) | 0.5163 | 0.0008 | 0.0240 |
| TG(46:3) | 0.5162 | 0.0008 | 0.0240 |
| TG(46:5) | 0.5156 | 0.0008 | 0.0240 |
| TG(47:5) | 0.5117 | 0.0009 | 0.0257 |
| TG(46:1) | 0.5066 | 0.0010 | 0.0282 |
| TG(46:2) | 0.5062 | 0.0010 | 0.0282 |
| TG(40:5) | 0.5052 | 0.0010 | 0.0283 |
| TG(48:8) | 0.5040 | 0.0011 | 0.0283 |
| TG(43:1) | 0.5025 | 0.0011 | 0.0283 |
| TG(40:3) | 0.5023 | 0.0011 | 0.0283 |
| TG(42:3) | 0.5023 | 0.0011 | 0.0283 |
| TG(46:8) | 0.5018 | 0.0011 | 0.0283 |
| TG(40:2) | 0.5011 | 0.0012 | 0.0283 |
| TG(44:4) | 0.5007 | 0.0012 | 0.0283 |
| TG(41:2) | 0.5002 | 0.0012 | 0.0283 |
| TG(48:7) | 0.4983 | 0.0012 | 0.0285 |
| TG(45:5) | 0.4663 | 0.0028 | 0.0407 |
| TG(55:1) | 0.4658 | 0.0028 | 0.0407 |
| TG(48:2) | 0.4630 | 0.0030 | 0.0423 |
| TG(51:0) | 0.4605 | 0.0032 | 0.0434 |
| TG(59:6) | 0.4605 | 0.0032 | 0.0434 |
| TG(49:7) | 0.4583 | 0.0033 | 0.0453 |
| TG(41:1) | 0.4579 | 0.0034 | 0.0454 |
| TG(50:6) | 0.4521 | 0.0039 | 0.0498 |
| **Phosphatidic acids** | | | |
| PA(36:1) | 0.5796 | 0.0001 | 0.0136 |
| **Lysophosphatidylcholines** | | | |
| LPC(14:1) | 0.4925 | 0.0014 | 0.0290 |
| LPC(15:1) | 0.4697 | 0.0026 | 0.0384 |
| **Diacylglycerols** | | | |
| DG(42:6) | 0.4869 | 0.0017 | 0.0316 |
| DG(28:1) | 0.4789 | 0.0020 | 0.0349 |
| DG(30:2) | 0.4676 | 0.0027 | 0.0400 |
| **Lysophosphatidylethanolamines** | | | |
| LPE(12:0) | 0.4807 | 0.0020 | 0.0344 |
| **Dihexosylceramides** | | | |
| Hex2Cer(d42:1) | 0.4763 | 0.0022 | 0.0361 |
| Hex2Cer(d40:1) | 0.5146 | 0.0008 | 0.0242 |
| Hex2Cer(d36:1) | 0.4568 | 0.0035 | 0.0461 |
| **Alkylacylglycerols** | | | |
| DG(O-42:1) | -0.4649 | 0.0029 | 0.0411 |
| **Hydroxysphingomyelins** | | | |
| SM(t40:1) | 0.4626 | 0.0030 | 0.0423 |
| **Alkyldiacylglycerols** | | | |
| TG(O-52:0) | 0.4561 | 0.0035 | 0.0462 |

**Table S6.** Plasma lipids that differ between individuals with MASLD without MASH and MASLD-free subjects. Shown are those lipids with an uncorrected p-value<0.005.

| Lipid | Log2(ratio) | p-value | FDR adjusted p-value | Order of abundancy |
| --- | --- | --- | --- | --- |
| PC(36:2) | 0.51978217 | 4.8262E-06 | 0.00778956 | 33 |
| PC(32:2) | 0.96402741 | 2.4221E-05 | 0.01954632 | 195 |
| LPC(15:1) | 1.29128198 | 2.5847E-05 | 0.01390558 | 1138 |
| PC(39:3) | 0.9308722 | 2.7647E-05 | 0.01115555 | 940 |
| PC(38:3) | 1.34621622 | 3.187E-05 | 0.01028772 | 104 |
| PC(34:5) | 1.24403228 | 4.8727E-05 | 0.0131076 | 875 |
| PC(32:4) | 1.47671965 | 6.0855E-05 | 0.01403135 | 1244 |
| PC(40:4) | 0.4860677 | 6.0911E-05 | 0.01228874 | 244 |
| CE(16:3) | 1.39017278 | 7.2429E-05 | 0.01298888 | 102 |
| CE(18:4) | 1.69512882 | 7.4655E-05 | 0.01204928 | 27 |
| PC(41:5) | 0.86395262 | 0.00010791 | 0.01583307 | 986 |
| PC(39:5) | 0.62173917 | 0.00012179 | 0.01638076 | 540 |
| CE(16:2) | 1.32370141 | 0.00015451 | 0.01918319 | 49 |
| PC(32:3) | 1.20155229 | 0.00018027 | 0.02078226 | 676 |
| PC(30:1) | 1.42854555 | 0.00020668 | 0.02223836 | 337 |
| PC(36:6) | 1.11104181 | 0.00025235 | 0.02545549 | 645 |
| LPC(13:0) | 0.65685645 | 0.00025697 | 0.02439721 | 1214 |
| PS(40:7) | 1.49930192 | 0.00026982 | 0.02419388 | 1156 |
| CE(18:3) | 0.98986868 | 0.00027465 | 0.02333121 | 5 |
| CE(17:2) | 1.17036016 | 0.00036313 | 0.02930496 | 74 |
| PC(29:1) | 2.63925813 | 0.00036983 | 0.02842437 | 1370 |
| SM(t29:1) | 1.43976099 | 0.00037027 | 0.02716437 | 1571 |
| PE(O-34:0) | 1.06398404 | 0.00040984 | 0.02876031 | 1358 |
| TG(49:1) | 1.56197337 | 0.00041063 | 0.02761492 | 211 |
| PC(28:1) | 1.95165169 | 0.0004126 | 0.0266374 | 934 |
| LPE(18:2) | 1.15187697 | 0.00041365 | 0.02567791 | 246 |
| TG(47:2) | 1.19125614 | 0.00042234 | 0.02524673 | 254 |
| PC(38:2) | 0.59752585 | 0.00042521 | 0.02451013 | 363 |
| TG(49:2) | 1.08703452 | 0.00042884 | 0.02386702 | 187 |
| Hex2Cer(d42:1) | 0.84793643 | 0.00043616 | 0.02346551 | 398 |
| PE(O-37:4) | 0.96138354 | 0.00046487 | 0.02420343 | 882 |
| PC(32:5) | 1.26418344 | 0.00047736 | 0.02407699 | 1477 |
| SPH(20:1) | 0.19961365 | 0.00051565 | 0.02522014 | 346 |
| SM(d40:1) | 0.42376185 | 0.00052804 | 0.02506635 | 54 |
| SM4(d38:1) | 1.15392008 | 0.00052805 | 0.0243508 | 1435 |
| PC(34:4) | 0.78612497 | 0.00055475 | 0.02487144 | 505 |
| TG(47:1) | 1.5206254 | 0.00057927 | 0.02526887 | 232 |
| PI(35:2) | 0.78071799 | 0.00059377 | 0.02521973 | 715 |
| TG(48:1) | 1.38700364 | 0.00060578 | 0.02506996 | 60 |
| TG(49:0) | 1.80184186 | 0.00063607 | 0.02566525 | 427 |
| TG(51:1) | 1.29965305 | 0.00063784 | 0.02510892 | 201 |
| PC(31:2) | 1.2065952 | 0.00068066 | 0.02615695 | 1276 |
| PC(30:2) | 1.60256387 | 0.00068609 | 0.02575236 | 769 |
| PS(40:6) | 1.18281144 | 0.00068664 | 0.02518712 | 523 |
| TG(48:0) | 1.63396188 | 0.00069575 | 0.02495427 | 132 |
| TG(54:0) | 1.19225256 | 0.00077767 | 0.02728612 | 320 |
| TG(53:0) | 0.92882926 | 0.00079352 | 0.02724972 | 576 |
| PC(34:0) | 0.5585061 | 0.00081789 | 0.02750165 | 353 |
| TG(O-48:1) | 1.65914969 | 0.00084314 | 0.02777211 | 1012 |
| SM(d41:1) | 0.43034997 | 0.00095052 | 0.03068285 | 110 |
| TG(47:3) | 1.21779342 | 0.00098833 | 0.03127759 | 379 |
| Hex2Cer(d40:1) | 0.84487162 | 0.00102556 | 0.03183185 | 392 |
| TG(46:1) | 1.77899475 | 0.00104849 | 0.03192947 | 86 |
| PC(42:6) | 0.70775595 | 0.00104983 | 0.03137836 | 410 |
| SM(d39:1) | 0.47531562 | 0.00106645 | 0.03129546 | 150 |
| TG(46:2) | 1.72574253 | 0.00110133 | 0.03174187 | 105 |
| TG(46:0) | 2.38183915 | 0.00113486 | 0.0321346 | 177 |
| TG(47:0) | 1.54356211 | 0.00113903 | 0.03169654 | 370 |
| CE(14:1) | 1.89581014 | 0.00116785 | 0.03194773 | 101 |
| SM(d38:1) | 0.31623204 | 0.00121701 | 0.03273768 | 73 |
| HexCer(d40:1) | 0.64966117 | 0.00121836 | 0.03223656 | 214 |
| PI(30:1) | 2.71206816 | 0.00123434 | 0.03213263 | 1286 |
| TG(44:2) | 2.64980931 | 0.0012587 | 0.03224672 | 210 |
| PC(37:1) | 0.76708455 | 0.00131195 | 0.03308583 | 985 |
| PC(33:1) | 0.65084286 | 0.00132236 | 0.03283522 | 263 |
| SM(d41:0) | 0.66480044 | 0.00134764 | 0.03295594 | 487 |
| SM(t32:3) | 1.39562173 | 0.00137584 | 0.03314339 | 1542 |
| PC(31:1) | 1.04693913 | 0.00142487 | 0.0338198 | 550 |
| TG(51:2) | 0.65701489 | 0.00143662 | 0.03360432 | 133 |
| CE(15:1) | 1.15713554 | 0.00145456 | 0.0335379 | 294 |
| CE(17:3) | 1.91877749 | 0.00145937 | 0.03317495 | 242 |
| TG(O-52:0) | 1.08134413 | 0.00146358 | 0.03280857 | 846 |
| PE(42:9) | 1.22525063 | 0.00148875 | 0.03291566 | 1300 |
| TG(O-53:3) | 1.30859524 | 0.00149224 | 0.03254688 | 1397 |
| TG(45:2) | 1.37517013 | 0.00150569 | 0.0324025 | 366 |
| PC(30:0) | 1.04059275 | 0.0015124 | 0.03211868 | 127 |
| TG(46:3) | 1.86001064 | 0.00151267 | 0.03170711 | 198 |
| TG(O-54:1) | 0.93976069 | 0.00155751 | 0.03222846 | 678 |
| TG(51:0) | 1.66389073 | 0.00158812 | 0.03244598 | 489 |
| Hex2Cer(d41:1) | 1.01116013 | 0.00158978 | 0.03207381 | 653 |
| Hex2Cer(d36:1) | 1.1427207 | 0.00160534 | 0.03198787 | 756 |
| PC(37:2) | 0.49531592 | 0.0016325 | 0.03213239 | 584 |
| SM4(d43:1) | 1.23881897 | 0.0016327 | 0.03174919 | 1587 |
| CE(16:1) | 0.95069902 | 0.00165211 | 0.03174402 | 10 |
| DG(28:1) | 1.83771505 | 0.00168203 | 0.03193875 | 606 |
| TG(47:4) | 1.60697377 | 0.00168565 | 0.03163526 | 610 |
| TG(44:1) | 2.44633457 | 0.00175978 | 0.03264696 | 139 |
| PC(28:2) | 2.1327055 | 0.00177804 | 0.03261087 | 1503 |
| HexCer(d42:1) | 0.65474991 | 0.00178268 | 0.03232855 | 171 |
| SM(d40:0) | 0.66206718 | 0.00178471 | 0.03200582 | 270 |
| PC(37:3) | 0.43852695 | 0.00185182 | 0.03284439 | 458 |
| PC(33:3) | 0.84282815 | 0.00193148 | 0.03388485 | 950 |
| PC(38:1) | 0.65678389 | 0.00202247 | 0.03509969 | 635 |
| PC(38:7) | 0.61918205 | 0.00207778 | 0.03567584 | 421 |
| LPC(O-18:2) | -0.6660815 | 0.00209266 | 0.03555324 | 596 |
| SM(d42:1) | 0.50102047 | 0.00213293 | 0.03585988 | #N/B |
| SM4(t36:1) | 0.7498997 | 0.00213503 | 0.03552521 | 1306 |
| HexCer(d44:1) | 0.60207401 | 0.00217517 | 0.03582378 | 895 |
| HexCer(d41:1) | 0.70308551 | 0.00221336 | 0.03608442 | 285 |
| TG(52:0) | 1.61220481 | 0.00226594 | 0.03657225 | 276 |
| Cer(d40:0) | 0.80131178 | 0.00236558 | 0.03780248 | 298 |
| Cer(d42:0) | 0.78781961 | 0.00239225 | 0.03785377 | 273 |
| TG(49:3) | 0.71055386 | 0.00240601 | 0.03770199 | 238 |
| TG(41:2) | 1.94499082 | 0.00242915 | 0.03769861 | 811 |
| TG(48:2) | 1.06344076 | 0.0024331 | 0.03740025 | 50 |
| TG(55:1) | 0.86884717 | 0.00248188 | 0.0377902 | 409 |
| TG(53:1) | 1.36162902 | 0.00252569 | 0.03809778 | 292 |
| LPC(14:1) | 1.66076008 | 0.00256101 | 0.03827281 | 1073 |
| PS(38:5) | 1.25711249 | 0.00264749 | 0.03920232 | 675 |
| SM(d32:0) | 0.54171552 | 0.00268115 | 0.03933972 | 472 |
| PI(36:5) | 1.36116253 | 0.00269238 | 0.03914863 | 1013 |
| LPE(12:0) | 1.00744505 | 0.0026941 | 0.03882392 | 1406 |
| TG(55:4) | 0.39378938 | 0.0027834 | 0.03975585 | 486 |
| PC(33:0) | 0.69235309 | 0.00282235 | 0.03995848 | 405 |
| PS(40:5) | 0.96847259 | 0.0028319 | 0.03974512 | 582 |
| LPC(O-23:1) | 0.85268278 | 0.00288126 | 0.04008923 | 1488 |
| TG(46:4) | 2.1540999 | 0.00291273 | 0.0401807 | 347 |
| TG(45:3) | 1.71118656 | 0.00301834 | 0.0412847 | 552 |
| PI(34:2) | 0.67725829 | 0.00312324 | 0.0423606 | 234 |
| HexCer(d38:1) | 0.58353619 | 0.00315755 | 0.0424691 | 494 |
| SM(d28:3) | 0.89221046 | 0.0031852 | 0.04248685 | 1530 |
| SM(d36:4) | 0.89420666 | 0.00320074 | 0.04234423 | 752 |
| TG(43:2) | 1.90268809 | 0.00326834 | 0.04288698 | 569 |
| HexCer(d42:0) | 0.74206443 | 0.00331352 | 0.04312921 | 1084 |
| TG(43:3) | 2.12434133 | 0.00333735 | 0.04309181 | 857 |
| PC(40:3) | 0.68907135 | 0.00334244 | 0.04281512 | 695 |
| CE(16:4) | 2.84625226 | 0.00337066 | 0.04283659 | 230 |
| TG(45:4) | 2.15844323 | 0.00345775 | 0.04360011 | 886 |
| SM(d42:0) | 0.75345846 | 0.00348951 | 0.04365939 | 521 |
| TG(44:3) | 2.58852413 | 0.00358973 | 0.0445679 | 357 |
| PC(32:1) | 0.61815005 | 0.00370527 | 0.04565118 | 89 |
| PE(40:4) | 0.78519583 | 0.00374161 | 0.04574966 | 525 |
| SM(d38:0) | 0.62208929 | 0.003793 | 0.04602929 | 381 |
| TG(45:1) | 1.28157799 | 0.00380666 | 0.04585038 | 291 |
| CE(17:1) | 0.81559683 | 0.00382295 | 0.04570551 | 16 |
| TG(50:8) | 1.53416185 | 0.00385297 | 0.0457257 | 775 |
| CE(14:0) | 1.73514253 | 0.00387895 | 0.04569798 | 63 |
| TG(42:2) | 2.51834816 | 0.00393059 | 0.04597082 | 330 |
| TG(48:6) | 2.11251305 | 0.00398612 | 0.04628483 | 506 |
| PS(38:4) | 1.23627151 | 0.00401932 | 0.04633697 | 220 |
| PC(36:1) | 0.52339161 | 0.00409493 | 0.04687389 | 144 |
| TG(O-51:0) | 0.73354393 | 0.00410288 | 0.04663419 | 1126 |
| PS(40:4) | 1.1852366 | 0.00417922 | 0.04716971 | 589 |
| PC(40:2) | 0.68789916 | 0.00420945 | 0.04718087 | 871 |
| TG(42:1) | 2.6908032 | 0.00422468 | 0.04702505 | 241 |
| PI(39:5) | 1.84037052 | 0.00425708 | 0.04706114 | 1311 |
| TG(51:8) | 1.68406029 | 0.00444882 | 0.04884625 | 1105 |
| PE(O-32:0) | 0.74618893 | 0.00454944 | 0.04961345 | 1413 |
| SM(d39:0) | 0.74934454 | 0.0045662 | 0.04946207 | 609 |
| TG(O-53:1) | 0.7381474 | 0.00468473 | >0.05 | 982 |
| PC(35:0) | 1.18498099 | 0.00468814 | >0.05 | 1122 |
| TG(O-55:2) | 0.60644505 | 0.00468873 | 0.04978696 | 1140 |
| TG(44:0) | 2.13883375 | 0.00472451 | 0.04983899 | 174 |
| LPE(16:1) | 0.99059949 | 0.00478337 | >0.05 | 810 |
| TG(48:7) | 2.12051131 | 0.00485032 | >0.05 | 750 |
| TG(O-52:1) | 1.02229979 | 0.00488495 | >0.05 | 493 |
| TG(45:0) | 1.2207433 | 0.0048856 | >0.05 | 358 |
| TG(49:5) | 1.22956461 | 0.00491837 | >0.05 | 604 |
| PI(32:1) | 1.32006157 | 0.00494687 | >0.05 | 473 |

**Table S7.** Plasma lipids that differ between individuals with MASLD whose have MASH and MASLD-free subjects. Shown are those lipids with an uncorrected p-value<0.005.

| Lipid | Log2(ratio) | p-value | FDR corrected | Order of abundancy |
| --- | --- | --- | --- | --- |
| TG(47:1) | 1.117964 | 2E-06 | 0.003258 | 232 |
| TG(46:0) | 2.122849 | 7.68E-06 | 0.006238 | 177 |
| TG(49:1) | 1.16169 | 1.35E-05 | 0.007339 | 211 |
| PC(29:1) | 2.104947 | 1.97E-05 | 0.008018 | 1370 |
| PC(30:1) | 1.469068 | 2.02E-05 | 0.006566 | 337 |
| TG(48:1) | 1.449017 | 4.58E-05 | 0.012402 | 60 |
| TG(47:0) | 0.869594 | 7.32E-05 | 0.017003 | 370 |
| TG(48:0) | 1.647393 | 8.34E-05 | 0.016935 | 132 |
| PC(28:1) | 1.583336 | 0.000104 | 0.018848 | 934 |
| PC(32:4) | 1.678804 | 0.000114 | 0.018595 | 1244 |
| Hex2Cer(d42:1) | 0.799233 | 0.000115 | 0.016926 | 398 |
| PC(32:2) | 0.80795 | 0.000133 | 0.017998 | 195 |
| LPC(13:0) | 0.415135 | 0.000165 | 0.020569 | 1214 |
| LPC(14:1) | 1.200396 | 0.000167 | 0.019385 | 1073 |
| TG(49:2) | 0.896802 | 0.000219 | 0.023774 | 187 |
| TG(46:1) | 1.849778 | 0.000239 | 0.024269 | 86 |
| PC(30:0) | 0.981943 | 0.000244 | 0.023291 | 127 |
| TG(44:0) | 1.899219 | 0.000328 | 0.029573 | 174 |
| TG(49:0) | 1.279106 | 0.000403 | 0.034491 | 427 |
| TG(47:2) | 1.084158 | 0.000428 | 0.034792 | 254 |
| PC(34:4) | 0.659093 | 0.000598 | 0.046252 | 505 |
| SM4(d38:1) | 0.922254 | 0.000705 | >0.05 | 1435 |
| SM(t30:2) | 0.881662 | 0.000707 | >0.05 | 1044 |
| TG(45:0) | 0.582675 | 0.000798 | >0.05 | 358 |
| TG(48:2) | 1.224558 | 0.000921 | >0.05 | 50 |
| PC(29:0) | 1.050993 | 0.001004 | >0.05 | 612 |
| CE(16:3) | 1.454628 | 0.00104 | >0.05 | 102 |
| HexCer(d42:0) | 0.791927 | 0.001069 | >0.05 | 1084 |
| TG(45:1) | 1.015151 | 0.001072 | >0.05 | 291 |
| LPE(22:4) | 0.966186 | 0.00127 | >0.05 | 466 |
| TG(51:2) | 0.540751 | 0.001402 | >0.05 | 133 |
| PI(34:3) | 0.967318 | 0.001413 | >0.05 | 899 |
| PC(32:3) | 1.093418 | 0.001547 | >0.05 | 676 |
| DG(O-42:8) | 0.899074 | 0.001551 | >0.05 | 781 |
| PC(34:5) | 1.085025 | 0.001569 | >0.05 | 875 |
| PI(30:1) | 2.32449 | 0.001637 | >0.05 | 1286 |
| TG(50:1) | 0.71686 | 0.00173 | >0.05 | 38 |
| PA(36:1) | 0.734917 | 0.001747 | >0.05 | 1271 |
| PC(31:2) | 1.254483 | 0.001772 | >0.05 | 1276 |
| LPC(23:2) | -1.24895 | 0.001875 | >0.05 | 1598 |
| LPE(12:0) | 0.966798 | 0.002025 | >0.05 | 1406 |
| PC(31:1) | 0.928149 | 0.002186 | >0.05 | 550 |
| SPH(17:1) | 0.14786 | 0.002336 | >0.05 | 79 |
| Hex2Cer(d40:1) | 0.733049 | 0.002605 | >0.05 | 392 |
| TG(62:10) | 1.456897 | 0.002724 | >0.05 | 881 |
| PC(39:3) | 0.772226 | 0.003109 | >0.05 | 940 |
| PI(34:2) | 0.714836 | 0.003138 | >0.05 | 234 |
| TG(O-48:1) | 1.301496 | 0.003353 | >0.05 | 1012 |
| PC(36:1) | 0.440307 | 0.00337 | >0.05 | 144 |
| TG(51:1) | 1.223348 | 0.003475 | >0.05 | 201 |
| LPC(15:1) | 0.83793 | 0.003495 | >0.05 | 1138 |
| TG(46:2) | 1.9715 | 0.003674 | >0.05 | 105 |
| SM(d38:4) | -0.75087 | 0.003711 | >0.05 | 868 |
| SM(t31:2) | 0.882702 | 0.003775 | >0.05 | 1293 |
| HexCer(d44:1) | 0.568055 | 0.0038 | >0.05 | 895 |
| SM4(d41:1) | 0.564906 | 0.003858 | >0.05 | 1112 |
| SM4(d40:1) | 0.58063 | 0.003909 | >0.05 | 887 |
| HexCer(d40:0) | 0.81831 | 0.004261 | >0.05 | 906 |
| PC(48:6) | 0.688475 | 0.004433 | >0.05 | 1439 |
| PC(42:6) | 0.720219 | 0.00455 | >0.05 | 410 |
| C1P(d50:1) | -0.8495 | 0.004669 | >0.05 | 812 |
| PI(35:2) | 0.73719 | 0.004866 | >0.05 | 715 |

**Table S8.** Plasma lipids that differ between MASLD individuals with and without MASH. Shown are those lipids with an uncorrected p-value<0.005.

| Lipid | Log2(ratio) | p-value | FDR corrected |  |
| --- | --- | --- | --- | --- |
| C1P(d45:1) | -1.347893728 | 0.002055857 | >0.05 | 1363 |
| PC(O-36:3) | -0.475597017 | 0.002604129 | >0.05 | 289 |
| PC(36:2) | -0.331087224 | 0.004518373 | >0.05 | 33 |
| PC(O-38:3) | -0.391053824 | 0.00488094 | >0.05 | 485 |

**Table S9.** Relative plasma abundancy of all detected lipids in all subjects, from high to low abundancy.

| Position | Lipid | Rel. abun. (%) |  | Position | Lipid | Rel. abun. (%) |
| --- | --- | --- | --- | --- | --- | --- |
| 1 | CE(18:2) | 44,40951 |  | 39 | TG(54:4) | 0,067746 |
| 2 | CE(20:4) | 16,77857 |  | 40 | LPC(18:0) | 0,067706 |
| 3 | CE(18:1) | 14,27721 |  | 41 | PC(38:4) | 0,065659 |
| 4 | CE(20:3) | 4,841415 |  | 42 | CE(22:1) | 0,058933 |
| 5 | CE(18:3) | 3,611707 |  | 43 | LPC(18:1) | 0,054672 |
| 6 | CE(20:5) | 3,170163 |  | 44 | TG(52:5) | 0,053355 |
| 7 | CE(22:6) | 2,158655 |  | 45 | CE(19:3) | 0,053248 |
| 8 | CE(16:0) | 1,239124 |  | 46 | TG(54:5) | 0,052694 |
| 9 | CE(18:0) | 0,993742 |  | 47 | LPC(20:4) | 0,049946 |
| 10 | CE(16:1) | 0,853322 |  | 48 | PC(36:3) | 0,046695 |
| 11 | CE(19:2) | 0,391987 |  | 49 | CE(16:2) | 0,043491 |
| 12 | CE(20:2) | 0,372835 |  | 50 | TG(48:2) | 0,043481 |
| 13 | CE(19:1) | 0,288966 |  | 51 | CE(24:1) | 0,043282 |
| 14 | CE(20:1) | 0,227377 |  | 52 | TG(54:6) | 0,043118 |
| 15 | LPC(16:0) | 0,202639 |  | 53 | CE(21:4) | 0,042508 |
| 16 | CE(17:1) | 0,202365 |  | 54 | SM(d40:1) | 0,041295 |
| 17 | PC(34:2) | 0,187331 |  | 55 | PC(38:6) | 0,03963 |
| 18 | DG(36:2) | 0,179466 |  | 56 | DG(36:4) | 0,036693 |
| 19 | TG(52:2) | 0,176995 |  | 57 | TG(54:2) | 0,035771 |
| 20 | TG(52:3) | 0,17567 |  | 58 | SM(d36:1) | 0,035668 |
| 21 | SM(d34:1) | 0,158865 |  | 59 | TG(50:4) | 0,035036 |
| 22 | CE(22:5) | 0,158223 |  | 60 | TG(48:1) | 0,034907 |
| 23 | DG(34:1) | 0,157217 |  | 61 | CE(22:3) | 0,033162 |
| 24 | CE(17:0) | 0,153543 |  | 62 | Cer(d43:1) | 0,033072 |
| 25 | TG(52:4) | 0,122439 |  | 63 | CE(14:0) | 0,032059 |
| 26 | DG(36:3) | 0,121201 |  | 64 | SM(d42:3) | 0,031441 |
| 27 | CE(18:4) | 0,119048 |  | 65 | PI(38:4) | 0,030996 |
| 28 | CE(22:4) | 0,112551 |  | 66 | PC(38:5) | 0,030355 |
| 29 | LPC(18:2) | 0,108806 |  | 67 | DG(32:0) | 0,029658 |
| 30 | PC(34:1) | 0,108113 |  | 68 | SM(d42:1) | 0,029385 |
| 31 | TG(50:2) | 0,094263 |  | 69 | PC(40:6) | 0,029235 |
| 32 | TG(50:3) | 0,075729 |  | 70 | DG(36:1) | 0,028211 |
| 33 | PC(36:2) | 0,075329 |  | 71 | SM(d40:2) | 0,027739 |
| 34 | TG(54:3) | 0,074888 |  | 72 | TG(52:1) | 0,027581 |
| 35 | DG(34:2) | 0,074363 |  | 73 | SM(d38:1) | 0,025931 |
| 36 | PC(36:4) | 0,072784 |  | 74 | CE(17:2) | 0,025621 |
| 37 | SM(d42:2) | 0,072043 |  | 75 | DG(32:1) | 0,024788 |
| 38 | TG(50:1) | 0,071237 |  | 76 | SM(d32:1) | 0,024625 |

| 77 | SM(d34:2) | 0,024453 |  | 115 | CE(24:6) | 0,011501 |
| --- | --- | --- | --- | --- | --- | --- |
| 78 | PC(32:0) | 0,024045 |  | 116 | PE(38:4) | 0,011353 |
| 79 | SPH(17:1) | 0,023698 |  | 117 | CE(15:0) | 0,010961 |
| 80 | TG(56:7) | 0,023591 |  | 118 | TG(56:5) | 0,010948 |
| 81 | TG(48:3) | 0,023508 |  | 119 | Cer(d42:2) | 0,010917 |
| 82 | CE(21:2) | 0,022761 |  | 120 | SM(d41:2) | 0,010744 |
| 83 | TG(54:7) | 0,022756 |  | 121 | CE(24:2) | 0,010627 |
| 84 | CE(21:5) | 0,022739 |  | 122 | CE(22:7) | 0,010571 |
| 85 | PC(O-38:5) | 0,021688 |  | 123 | PE(O-38:6) | 0,010381 |
| 86 | TG(46:1) | 0,021071 |  | 124 | TG(50:5) | 0,010336 |
| 87 | LPC(22:6) | 0,021033 |  | 125 | CE(30:0) | 0,010058 |
| 88 | Cer(d42:1) | 0,020873 |  | 126 | SM(d33:1) | 0,010048 |
| 89 | PC(32:1) | 0,019971 |  | 127 | PC(30:0) | 0,009801 |
| 90 | DG(38:5) | 0,019629 |  | 128 | CE(23:1) | 0,009239 |
| 91 | CE(24:4) | 0,019478 |  | 129 | CE(25:1) | 0,009169 |
| 92 | CE(22:2) | 0,019154 |  | 130 | CE(26:1) | 0,009135 |
| 93 | CE(20:0) | 0,019036 |  | 131 | SM(d38:2) | 0,008792 |
| 94 | CE(19:0) | 0,018845 |  | 132 | TG(48:0) | 0,00872 |
| 95 | TG(56:8) | 0,018801 |  | 133 | TG(51:2) | 0,008694 |
| 96 | DG(34:3) | 0,018744 |  | 134 | CE(25:0) | 0,008677 |
| 97 | CE(24:0) | 0,018202 |  | 135 | PC(O-44:5) | 0,008622 |
| 98 | TG(56:6) | 0,017924 |  | 136 | PC(O-36:4) | 0,008545 |
| 99 | SM(d36:2) | 0,017134 |  | 137 | DG(36:5) | 0,008481 |
| 100 | TG(52:6) | 0,017082 |  | 138 | PC(36:5) | 0,008312 |
| 101 | CE(14:1) | 0,016455 |  | 139 | TG(44:1) | 0,008221 |
| 102 | CE(16:3) | 0,016264 |  | 140 | PC(O-38:4) | 0,008089 |
| 103 | DG(34:0) | 0,016185 |  | 141 | PI(36:2) | 0,008042 |
| 104 | PC(38:3) | 0,01573 |  | 142 | LPE(20:4) | 0,007933 |
| 105 | TG(46:2) | 0,01509 |  | 143 | Hex2Cer(d34:1) | 0,007619 |
| 106 | CE(19:4) | 0,014653 |  | 144 | PC(36:1) | 0,007559 |
| 107 | CE(22:0) | 0,014395 |  | 145 | TG(56:9) | 0,007437 |
| 108 | DG(38:6) | 0,014048 |  | 146 | DG(30:0) | 0,007424 |
| 109 | PE(O-38:5) | 0,013326 |  | 147 | PC(40:5) | 0,007357 |
| 110 | SM(d41:1) | 0,012985 |  | 148 | DG(40:7) | 0,007273 |
| 111 | DG(38:4) | 0,012984 |  | 149 | PE(O-36:5) | 0,007118 |
| 112 | CE(21:3) | 0,01297 |  | 150 | SM(d39:1) | 0,007069 |
| 113 | CE(24:5) | 0,012178 |  | 151 | CE(26:0) | 0,006775 |
| 114 | CE(21:1) | 0,011701 |  | 152 | CE(24:3) | 0,006719 |

| 153 | TG(53:2) | 0,006671 |  | 191 | TG(58:10) | 0,00467 |
| --- | --- | --- | --- | --- | --- | --- |
| 154 | TG(48:4) | 0,006662 |  | 192 | TG(56:4) | 0,004597 |
| 155 | LPC(16:1) | 0,006656 |  | 193 | PE(40:6) | 0,004591 |
| 156 | CE(23:0) | 0,006643 |  | 194 | DG(40:8) | 0,004544 |
| 157 | LPC(22:5) | 0,006609 |  | 195 | PC(32:2) | 0,004501 |
| 158 | TG(56:3) | 0,00648 |  | 196 | TG(58:2) | 0,004422 |
| 159 | DG(32:2) | 0,00634 |  | 197 | DG(38:2) | 0,004342 |
| 160 | TG(51:3) | 0,006284 |  | 198 | TG(46:3) | 0,004295 |
| 161 | Cer(d40:1) | 0,006252 |  | 199 | CE(21:0) | 0,004287 |
| 162 | TG(54:8) | 0,006219 |  | 200 | TG(58:8) | 0,004225 |
| 163 | PE(O-40:7) | 0,006173 |  | 201 | TG(51:1) | 0,004221 |
| 164 | PE(36:2) | 0,006134 |  | 202 | DG(35:2) | 0,004208 |
| 165 | SM(d34:0) | 0,005956 |  | 203 | SM(d42:1) | 0,004143 |
| 166 | PC(O-40:7) | 0,00575 |  | 204 | CE(26:2) | 0,004102 |
| 167 | PC(O-40:6) | 0,005728 |  | 205 | LPC(20:5) | 0,004088 |
| 168 | TG(54:1) | 0,005708 |  | 206 | DG(35:1) | 0,004028 |
| 169 | DG(40:6) | 0,005666 |  | 207 | SM(d35:1) | 0,004013 |
| 170 | TG(50:0) | 0,005586 |  | 208 | HexCer(d42:2) | 0,003884 |
| 171 | HexCer(d42:1) | 0,005558 |  | 209 | TG(52:7) | 0,003864 |
| 172 | PC(40:7) | 0,005535 |  | 210 | TG(44:2) | 0,003831 |
| 173 | Cer(d41:1) | 0,005458 |  | 211 | TG(49:1) | 0,003693 |
| 174 | TG(44:0) | 0,005457 |  | 212 | TG(58:3) | 0,003665 |
| 175 | PC(O-38:6) | 0,00543 |  | 213 | PE(36:4) | 0,003654 |
| 176 | TG(53:3) | 0,005389 |  | 214 | HexCer(d40:1) | 0,003635 |
| 177 | TG(46:0) | 0,005348 |  | 215 | CE(26:5) | 0,003618 |
| 178 | PC(O-42:5) | 0,005347 |  | 216 | PI(36:4) | 0,003614 |
| 179 | PC(34:3) | 0,005296 |  | 217 | DG(33:1) | 0,003472 |
| 180 | PE(O-38:7) | 0,005213 |  | 218 | TG(51:4) | 0,003471 |
| 181 | PC(O-40:5) | 0,005161 |  | 219 | PI(34:1) | 0,003467 |
| 182 | CE(26:4) | 0,00516 |  | 220 | PS(38:4) | 0,003466 |
| 183 | PE(38:6) | 0,005126 |  | 221 | PI(38:3) | 0,003449 |
| 184 | DG(38:3) | 0,005103 |  | 222 | PE(O-40:8) | 0,00344 |
| 185 | TG(58:9) | 0,005081 |  | 223 | CE(26:3) | 0,003436 |
| 186 | LPE(22:6) | 0,005063 |  | 224 | TG(56:1) | 0,00336 |
| 187 | TG(49:2) | 0,004929 |  | 225 | TG(58:7) | 0,003355 |
| 188 | DG(36:0) | 0,004861 |  | 226 | PC(O-36:5) | 0,00322 |
| 189 | PC(O-44:6) | 0,004796 |  | 227 | SM(d43:2) | 0,003134 |
| 190 | TG(56:2) | 0,00471 |  | 228 | PC(O-42:6) | 0,003093 |

| 229 | PE(38:5) | 0,003036 |  | 267 | SM(d40:3) | 0,00214 |
| --- | --- | --- | --- | --- | --- | --- |
| 230 | CE(16:4) | 0,003016 |  | 268 | TG(58:1) | 0,002091 |
| 231 | PE(O-40:6) | 0,00291 |  | 269 | PC(O-42:4) | 0,002089 |
| 232 | TG(47:1) | 0,002901 |  | 270 | SM(d40:0) | 0,00204 |
| 233 | TG(53:4) | 0,002887 |  | 271 | PC(O-34:2) | 0,002029 |
| 234 | PI(34:2) | 0,002871 |  | 272 | PE(O-36:4) | 0,002015 |
| 235 | LPC(20:3) | 0,00287 |  | 273 | Cer(d42:0) | 0,002001 |
| 236 | PI(36:1) | 0,002837 |  | 274 | TG(58:6) | 0,00199 |
| 237 | DG(38:7) | 0,002834 |  | 275 | LPC(15:0) | 0,001986 |
| 238 | TG(49:3) | 0,002797 |  | 276 | TG(52:0) | 0,001978 |
| 239 | DG(30:1) | 0,002791 |  | 277 | TG(56:10) | 0,001967 |
| 240 | LPC(17:0) | 0,002766 |  | 278 | DG(34:4) | 0,001958 |
| 241 | TG(42:1) | 0,002766 |  | 279 | PC(31:0) | 0,001937 |
| 242 | CE(17:3) | 0,002759 |  | 280 | TG(60:2) | 0,001932 |
| 243 | SM(d42:4) | 0,002753 |  | 281 | SM(d32:2) | 0,001922 |
| 244 | PC(40:4) | 0,002702 |  | 282 | PC(O-44:7) | 0,001899 |
| 245 | CE(30:1) | 0,002681 |  | 283 | DG(33:2) | 0,001895 |
| 246 | LPE(18:2) | 0,00266 |  | 284 | PC(O-46:7) | 0,001879 |
| 247 | CE(23:2) | 0,002641 |  | 285 | HexCer(d41:1) | 0,001863 |
| 248 | TG(58:11) | 0,00264 |  | 286 | TG(53:5) | 0,001825 |
| 249 | PE(34:2) | 0,002627 |  | 287 | TG(60:3) | 0,001816 |
| 250 | CE(28:1) | 0,002625 |  | 288 | PC(33:2) | 0,001812 |
| 251 | DG(40:5) | 0,002609 |  | 289 | PC(O-36:3) | 0,001811 |
| 252 | TG(50:6) | 0,002594 |  | 290 | DG(33:0) | 0,001808 |
| 253 | CE(27:1) | 0,002547 |  | 291 | TG(45:1) | 0,001807 |
| 254 | TG(47:2) | 0,002541 |  | 292 | TG(53:1) | 0,001798 |
| 255 | SM(d37:1) | 0,002531 |  | 293 | CE(32:1) | 0,00177 |
| 256 | PC(O-40:4) | 0,002518 |  | 294 | CE(15:1) | 0,001746 |
| 257 | Hex2Cer(d42:2) | 0,002508 |  | 295 | LPC(22:4) | 0,001733 |
| 258 | PE(O-36:3) | 0,002469 |  | 296 | PE(34:1) | 0,001719 |
| 259 | PC(35:2) | 0,002452 |  | 297 | PS(36:1) | 0,001712 |
| 260 | CE(23:3) | 0,00245 |  | 298 | Cer(d40:0) | 0,001696 |
| 261 | CE(27:0) | 0,002425 |  | 299 | LPC(O-18:1) | 0,001667 |
| 262 | CE(25:2) | 0,002387 |  | 300 | CE(34:4) | 0,00166 |
| 263 | PC(33:1) | 0,002322 |  | 301 | CE(26:6) | 0,001658 |
| 264 | PC(O-32:1) | 0,002312 |  | 302 | SM(d36:0) | 0,001642 |
| 265 | LPC(O-16:0) | 0,00218 |  | 303 | TG(58:4) | 0,001639 |
| 266 | PE(O-40:5) | 0,002148 |  | 304 | TG(60:12) | 0,001638 |

| 305 | CE(34:3) | 0,001622 |  | 343 | LPE(16:0) | 0,001172 |
| --- | --- | --- | --- | --- | --- | --- |
| 306 | PC(40:8) | 0,001618 |  | 344 | TG(53:6) | 0,001157 |
| 307 | TG(40:0) | 0,001616 |  | 345 | LPE(22:5) | 0,001138 |
| 308 | LPC(20:2) | 0,001604 |  | 346 | SPH(20:1) | 0,001134 |
| 309 | PE(36:3) | 0,001599 |  | 347 | TG(46:4) | 0,001134 |
| 310 | TG(48:5) | 0,001593 |  | 348 | LPC(20:1) | 0,001126 |
| 311 | CE(27:2) | 0,001591 |  | 349 | CE(25:4) | 0,001125 |
| 312 | SM(t34:1) | 0,001542 |  | 350 | Cer(d38:1) | 0,001118 |
| 313 | DG(31:0) | 0,001534 |  | 351 | TG(54:9) | 0,001114 |
| 314 | PE(O-34:3) | 0,001527 |  | 352 | SM(d39:2) | 0,001103 |
| 315 | DG(40:2) | 0,00152 |  | 353 | PC(34:0) | 0,001099 |
| 316 | PI(38:5) | 0,001514 |  | 354 | SM(d36:3) | 0,001086 |
| 317 | PI(36:3) | 0,00151 |  | 355 | SM(d41:3) | 0,001081 |
| 318 | TG(40:1) | 0,001487 |  | 356 | TG(43:0) | 0,00107 |
| 319 | DG(38:1) | 0,00144 |  | 357 | TG(44:3) | 0,001068 |
| 320 | TG(54:0) | 0,001434 |  | 358 | TG(45:0) | 0,001055 |
| 321 | Cer(d42:3) | 0,001429 |  | 359 | DG(35:3) | 0,001055 |
| 322 | TG(51:5) | 0,001402 |  | 360 | Cer(d41:2) | 0,001047 |
| 323 | LPE(18:1) | 0,001381 |  | 361 | DG(31:1) | 0,001036 |
| 324 | PC(37:4) | 0,001375 |  | 362 | PE(40:5) | 0,001028 |
| 325 | CE(28:0) | 0,001362 |  | 363 | PC(38:2) | 0,00102 |
| 326 | PC(O-32:0) | 0,001354 |  | 364 | Cer(d43:2) | 0,001013 |
| 327 | LPE(18:0) | 0,001349 |  | 365 | LPC(18:3) | 0,001012 |
| 328 | LPC(O-16:1) | 0,001337 |  | 366 | TG(45:2) | 0,001004 |
| 329 | PC(O-34:1) | 0,00133 |  | 367 | PC(35:1) | 0,000964 |
| 330 | TG(42:2) | 0,001314 |  | 368 | PI(40:6) | 0,000961 |
| 331 | PC(O-42:7) | 0,001309 |  | 369 | TG(60:4) | 0,000953 |
| 332 | SM(d43:1) | 0,001293 |  | 370 | TG(47:0) | 0,000943 |
| 333 | CE(14:2) | 0,001292 |  | 371 | TG(38:0) | 0,000923 |
| 334 | DG(26:0) | 0,001281 |  | 372 | DG(40:1) | 0,000923 |
| 335 | PE(36:1) | 0,001261 |  | 373 | TG(58:5) | 0,00091 |
| 336 | Cer(d40:2) | 0,001227 |  | 374 | CE(32:3) | 0,000902 |
| 337 | PC(30:1) | 0,001224 |  | 375 | TG(55:2) | 0,000894 |
| 338 | TG(60:11) | 0,001213 |  | 376 | TG(49:4) | 0,000889 |
| 339 | PE(O-38:4) | 0,00121 |  | 377 | DG(40:3) | 0,000882 |
| 340 | DG(O-42:7) | 0,0012 |  | 378 | PC(O-46:8) | 0,000882 |
| 341 | TG(38:1) | 0,001196 |  | 379 | TG(47:3) | 0,000872 |
| 342 | PC(O-46:6) | 0,001173 |  | 380 | DG(36:6) | 0,00086 |

| 381 | SM(d38:0) | 0,000859 |  | 419 | PI(40:5) | 0,0007 |
| --- | --- | --- | --- | --- | --- | --- |
| 382 | PC(42:10) | 0,000853 |  | 420 | TG(55:3) | 0,000699 |
| 383 | PC(O-44:4) | 0,000851 |  | 421 | PC(38:7) | 0,000696 |
| 384 | PE(O-34:2) | 0,00085 |  | 422 | PE(O-36:2) | 0,000687 |
| 385 | Cer(d34:1) | 0,000831 |  | 423 | TG(62:3) | 0,000686 |
| 386 | CE(32:2) | 0,000829 |  | 424 | TG(55:5) | 0,000685 |
| 387 | PE(38:3) | 0,000825 |  | 425 | CE(36:4) | 0,000679 |
| 388 | TG(57:2) | 0,000822 |  | 426 | Cer(d39:1) | 0,000676 |
| 389 | PC(O-44:8) | 0,00082 |  | 427 | TG(49:0) | 0,000672 |
| 390 | TG(56:0) | 0,000818 |  | 428 | LPE(20:5) | 0,000672 |
| 391 | TG(55:7) | 0,000818 |  | 429 | DG(40:4) | 0,000667 |
| 392 | Hex2Cer(d40:1) | 0,000813 |  | 430 | TG(43:1) | 0,000667 |
| 393 | PC(O-38:7) | 0,000806 |  | 431 | PE(O-36:6) | 0,000657 |
| 394 | DG(32:3) | 0,000799 |  | 432 | TG(59:2) | 0,000657 |
| 395 | DG(37:2) | 0,000788 |  | 433 | HexCer(d35:1) | 0,000652 |
| 396 | TG(58:12) | 0,000785 |  | 434 | CE(34:2) | 0,00064 |
| 397 | TG(55:6) | 0,000785 |  | 435 | PE(O-37:5) | 0,00064 |
| 398 | Hex2Cer(d42:1) | 0,000783 |  | 436 | CE(34:5) | 0,000639 |
| 399 | PE(40:7) | 0,000782 |  | 437 | TG(41:0) | 0,000639 |
| 400 | CE(25:3) | 0,000776 |  | 438 | DG(42:10) | 0,000635 |
| 401 | TG(36:0) | 0,000771 |  | 439 | HexCer(d40:2) | 0,000627 |
| 402 | TG(57:1) | 0,000761 |  | 440 | SM(d43:3) | 0,000627 |
| 403 | Cer(d41:0) | 0,00076 |  | 441 | PC(O-46:5) | 0,000625 |
| 404 | LPC(O-24:1) | 0,000758 |  | 442 | CE(28:2) | 0,00062 |
| 405 | PC(33:0) | 0,000755 |  | 443 | CE(29:1) | 0,00062 |
| 406 | CE(32:4) | 0,00075 |  | 444 | PE(O-42:7) | 0,000616 |
| 407 | PC(39:6) | 0,000746 |  | 445 | SM(d31:1) | 0,000615 |
| 408 | TG(52:8) | 0,000742 |  | 446 | LPE(O-18:1) | 0,000608 |
| 409 | TG(55:1) | 0,000742 |  | 447 | Hex2Cer(d42:3) | 0,000608 |
| 410 | PC(42:6) | 0,000741 |  | 448 | LPA(16:0) | 0,000605 |
| 411 | DG(42:7) | 0,000739 |  | 449 | PC(O-40:8) | 0,000602 |
| 412 | PC(O-36:2) | 0,000732 |  | 450 | SM(d35:2) | 0,000599 |
| 413 | TG(60:13) | 0,000729 |  | 451 | PC(O-42:3) | 0,000587 |
| 414 | SM(d44:2) | 0,000725 |  | 452 | LPA(18:2) | 0,000583 |
| 415 | TG(60:1) | 0,000724 |  | 453 | PC(42:7) | 0,000579 |
| 416 | CE(29:0) | 0,00072 |  | 454 | LPC(O-24:0) | 0,000577 |
| 417 | Hex2Cer(d32:1) | 0,000715 |  | 455 | SM(t42:1) | 0,000575 |
| 418 | TG(60:10) | 0,000704 |  | 456 | DG(42:6) | 0,000572 |

| 457 | SM(t42:2) | 0,000569 |  | 495 | LPE(20:3) | 0,000465 |
| --- | --- | --- | --- | --- | --- | --- |
| 458 | PC(37:3) | 0,000553 |  | 496 | LPC(O-18:0) | 0,000463 |
| 459 | HexCer(d42:3) | 0,000548 |  | 497 | PE(O-42:6) | 0,000463 |
| 460 | SPH(18:1) | 0,000545 |  | 498 | TG(59:1) | 0,000461 |
| 461 | TG(62:2) | 0,000544 |  | 499 | CE(27:3) | 0,00046 |
| 462 | LPC(20:0) | 0,000534 |  | 500 | SM(d38:3) | 0,000457 |
| 463 | PC(26:0) | 0,000524 |  | 501 | TG(59:3) | 0,000451 |
| 464 | Cer(d36:1) | 0,000523 |  | 502 | CE(30:2) | 0,000445 |
| 465 | TG(40:2) | 0,000521 |  | 503 | DG(29:0) | 0,000444 |
| 466 | LPE(22:4) | 0,00052 |  | 504 | CE(34:1) | 0,000443 |
| 467 | CE(28:5) | 0,00052 |  | 505 | PC(34:4) | 0,000439 |
| 468 | TG(50:7) | 0,000519 |  | 506 | TG(48:6) | 0,000435 |
| 469 | SM(d44:3) | 0,000505 |  | 507 | SM(d42:5) | 0,000434 |
| 470 | LPC(17:1) | 0,000503 |  | 508 | TG(60:9) | 0,000432 |
| 471 | TG(O-52:2) | 0,0005 |  | 509 | TG(51:6) | 0,000432 |
| 472 | SM(d32:0) | 0,000499 |  | 510 | PC(O-42:8) | 0,000432 |
| 473 | PI(32:1) | 0,000499 |  | 511 | PI(38:6) | 0,000429 |
| 474 | TG(53:7) | 0,000497 |  | 512 | TG(55:0) | 0,000429 |
| 475 | DG(30:2) | 0,000496 |  | 513 | CE(28:4) | 0,000428 |
| 476 | Cer(d44:1) | 0,000495 |  | 514 | DG(35:0) | 0,000426 |
| 477 | LPC(12:0) | 0,000493 |  | 515 | CE(31:1) | 0,000425 |
| 478 | PC(42:8) | 0,000493 |  | 516 | TG(42:3) | 0,000424 |
| 479 | PC(42:9) | 0,000489 |  | 517 | LPE(O-16:1) | 0,000423 |
| 480 | CE(36:5) | 0,000489 |  | 518 | TG(56:11) | 0,000421 |
| 481 | SM(t34:0) | 0,000489 |  | 519 | Cer(d44:2) | 0,000415 |
| 482 | PC(42:5) | 0,000488 |  | 520 | DG(40:0) | 0,000411 |
| 483 | TG(O-50:1) | 0,000483 |  | 521 | SM(d42:0) | 0,000411 |
| 484 | CE(28:3) | 0,000482 |  | 522 | TG(38:2) | 0,00041 |
| 485 | PC(O-38:3) | 0,000481 |  | 523 | PS(40:6) | 0,00041 |
| 486 | TG(55:4) | 0,000479 |  | 524 | TG(58:0) | 0,000405 |
| 487 | SM(d41:0) | 0,000478 |  | 525 | PE(40:4) | 0,000394 |
| 488 | DG(37:3) | 0,000477 |  | 526 | SM(t34:2) | 0,000391 |
| 489 | TG(51:0) | 0,000477 |  | 527 | DG(38:0) | 0,000389 |
| 490 | SM(d37:2) | 0,000475 |  | 528 | DG(42:8) | 0,000387 |
| 491 | SM4(t34:1) | 0,000474 |  | 529 | DG(45:7) | 0,000386 |
| 492 | TG(55:8) | 0,000472 |  | 530 | TG(44:4) | 0,000379 |
| 493 | TG(O-52:1) | 0,000465 |  | 531 | TG(57:0) | 0,000375 |
| 494 | HexCer(d38:1) | 0,000465 |  | 532 | TG(34:0) | 0,000374 |

| 533 | DG(37:4) | 0,000367 |  | 571 | C1P(d48:1) | 0,000292 |
| --- | --- | --- | --- | --- | --- | --- |
| 534 | DG(42:2) | 0,000366 |  | 572 | CE(30:3) | 0,00029 |
| 535 | HexCer(d43:1) | 0,000357 |  | 573 | SM(t40:1) | 0,000287 |
| 536 | TG(62:4) | 0,000356 |  | 574 | PE(O-42:8) | 0,000287 |
| 537 | TG(36:1) | 0,000355 |  | 575 | DG(42:9) | 0,000285 |
| 538 | TG(62:14) | 0,000351 |  | 576 | TG(53:0) | 0,000282 |
| 539 | LPC(24:0) | 0,000347 |  | 577 | TG(61:2) | 0,000281 |
| 540 | PC(39:5) | 0,000345 |  | 578 | Cer(d38:0) | 0,000279 |
| 541 | LPE(O-18:2) | 0,000345 |  | 579 | TG(60:5) | 0,000277 |
| 542 | SM4(d34:1) | 0,000342 |  | 580 | DG(39:0) | 0,000276 |
| 543 | PE(O-39:7) | 0,00034 |  | 581 | SM(t36:1) | 0,000276 |
| 544 | PC(O-40:3) | 0,00034 |  | 582 | PS(40:5) | 0,000273 |
| 545 | DG(40:9) | 0,000339 |  | 583 | DG(37:1) | 0,000273 |
| 546 | TG(62:13) | 0,000337 |  | 584 | PC(37:2) | 0,000272 |
| 547 | TG(60:7) | 0,000336 |  | 585 | DG(35:4) | 0,000271 |
| 548 | TG(60:8) | 0,000336 |  | 586 | PI(37:4) | 0,00027 |
| 549 | PE(O-42:5) | 0,000336 |  | 587 | TG(O-54:2) | 0,000268 |
| 550 | PC(31:1) | 0,000333 |  | 588 | DG(41:0) | 0,000266 |
| 551 | DG(42:1) | 0,00033 |  | 589 | PS(40:4) | 0,000265 |
| 552 | TG(45:3) | 0,000324 |  | 590 | DG(41:5) | 0,000264 |
| 553 | TG(46:5) | 0,000318 |  | 591 | TG(54:10) | 0,000262 |
| 554 | Hex2Cer(d40:2) | 0,000317 |  | 592 | LPC(O-22:0) | 0,000261 |
| 555 | TG(57:7) | 0,000317 |  | 593 | PC(O-32:2) | 0,000259 |
| 556 | PC(O-37:4) | 0,000317 |  | 594 | PE(36:5) | 0,000258 |
| 557 | TG(57:8) | 0,000307 |  | 595 | SM(d40:4) | 0,000257 |
| 558 | PC(O-37:5) | 0,000307 |  | 596 | LPC(O-18:2) | 0,000257 |
| 559 | PC(39:4) | 0,000306 |  | 597 | DG(O-34:1) | 0,000254 |
| 560 | TG(60:6) | 0,000306 |  | 598 | DG(37:0) | 0,000253 |
| 561 | DG(37:5) | 0,000305 |  | 599 | DG(24:0) | 0,000248 |
| 562 | SM(d33:2) | 0,000305 |  | 600 | TG(57:3) | 0,000248 |
| 563 | PC(O-30:1) | 0,000302 |  | 601 | PC(35:4) | 0,000247 |
| 564 | LPC(O-22:1) | 0,000302 |  | 602 | LPC(19:0) | 0,000247 |
| 565 | PC(O-42:2) | 0,0003 |  | 603 | CE(32:5) | 0,000247 |
| 566 | HexCer(d41:2) | 0,000298 |  | 604 | TG(49:5) | 0,000247 |
| 567 | PC(37:5) | 0,000297 |  | 605 | LPC(O-24:2) | 0,00024 |
| 568 | CE(36:3) | 0,000292 |  | 606 | DG(28:1) | 0,000234 |
| 569 | TG(43:2) | 0,000292 |  | 607 | SM(d42:6) | 0,000234 |
| 570 | PC(O-30:0) | 0,000292 |  | 608 | HexCer(d39:1) | 0,000232 |

| 609 | SM(d39:0) | 0,000231 |  | 647 | DG(39:3) | 0,000181 |
| --- | --- | --- | --- | --- | --- | --- |
| 610 | TG(47:4) | 0,00023 |  | 648 | LPC(24:6) | 0,000181 |
| 611 | DG(34:5) | 0,000226 |  | 649 | PC(O-46:9) | 0,000181 |
| 612 | PC(29:0) | 0,000223 |  | 650 | PC(44:5) | 0,00018 |
| 613 | HexCer(d43:2) | 0,000221 |  | 651 | SM(t42:3) | 0,000178 |
| 614 | PE(O-44:6) | 0,00022 |  | 652 | PC(O-36:6) | 0,000177 |
| 615 | PC(35:3) | 0,00022 |  | 653 | Hex2Cer(d41:1) | 0,000175 |
| 616 | DG(44:12) | 0,00022 |  | 654 | C1P(d47:1) | 0,000174 |
| 617 | PI(40:4) | 0,000218 |  | 655 | Cer(d27:1) | 0,000174 |
| 618 | LPA(20:4) | 0,000215 |  | 656 | DG(44:10) | 0,000174 |
| 619 | TG(57:6) | 0,000213 |  | 657 | LPC(22:0) | 0,000173 |
| 620 | PE(O-40:4) | 0,000212 |  | 658 | PC(41:6) | 0,000173 |
| 621 | Cer(d32:1) | 0,000211 |  | 659 | SM(d28:1) | 0,000173 |
| 622 | DG(39:6) | 0,000208 |  | 660 | HexCer(d36:1) | 0,000172 |
| 623 | TG(41:1) | 0,000207 |  | 661 | PE(O-44:7) | 0,000172 |
| 624 | PE(O-38:3) | 0,000206 |  | 662 | Cer(d43:0) | 0,000171 |
| 625 | Cer(d44:0) | 0,000205 |  | 663 | TG(64:3) | 0,000171 |
| 626 | Cer(d37:1) | 0,000202 |  | 664 | LPC(19:1) | 0,000169 |
| 627 | TG(62:1) | 0,000201 |  | 665 | PE(O-34:1) | 0,000169 |
| 628 | DG(O-35:3) | 0,0002 |  | 666 | Cer(d36:0) | 0,000169 |
| 629 | PS(36:2) | 0,000199 |  | 667 | LPC(24:5) | 0,000167 |
| 630 | TG(61:3) | 0,000199 |  | 668 | Cer(d45:1) | 0,000166 |
| 631 | PE(O-39:6) | 0,000198 |  | 669 | TG(52:9) | 0,000166 |
| 632 | LPA(18:1) | 0,000197 |  | 670 | PE(O-39:5) | 0,000165 |
| 633 | SM(t32:1) | 0,000195 |  | 671 | TG(64:2) | 0,000163 |
| 634 | PC(42:4) | 0,000194 |  | 672 | TG(61:1) | 0,000162 |
| 635 | PC(38:1) | 0,000194 |  | 673 | SM4(t42:2) | 0,000161 |
| 636 | TG(57:9) | 0,000193 |  | 674 | TG(62:6) | 0,00016 |
| 637 | DG(33:3) | 0,000193 |  | 675 | PS(38:5) | 0,000159 |
| 638 | DG(39:5) | 0,000192 |  | 676 | PC(32:3) | 0,000159 |
| 639 | SM(d44:1) | 0,000192 |  | 677 | TG(62:12) | 0,000158 |
| 640 | DG(42:0) | 0,000191 |  | 678 | TG(O-54:1) | 0,000158 |
| 641 | TG(62:5) | 0,000191 |  | 679 | LPC(O-22:2) | 0,000158 |
| 642 | TG(O-58:2) | 0,000189 |  | 680 | PC(37:6) | 0,000158 |
| 643 | TG(O-58:3) | 0,000187 |  | 681 | LPA(18:0) | 0,000158 |
| 644 | TG(O-56:2) | 0,000186 |  | 682 | PC(O-40:2) | 0,000157 |
| 645 | PC(36:6) | 0,000183 |  | 683 | LPC(22:1) | 0,000157 |
| 646 | PI(32:0) | 0,000182 |  | 684 | LPC(O-20:0) | 0,000156 |

| 685 | PC(44:4) | 0,000155 |  | 723 | TG(59:4) | 0,000131 |
| --- | --- | --- | --- | --- | --- | --- |
| 686 | TG(55:9) | 0,000154 |  | 724 | SM(d34:3) | 0,000131 |
| 687 | DG(39:4) | 0,000151 |  | 725 | DG(42:3) | 0,000131 |
| 688 | TG(32:0) | 0,000149 |  | 726 | LPC(O-16:2) | 0,000131 |
| 689 | DG(42:5) | 0,000149 |  | 727 | TG(60:0) | 0,00013 |
| 690 | PE(O-44:8) | 0,000149 |  | 728 | TG(O-56:1) | 0,000129 |
| 691 | TG(59:0) | 0,000148 |  | 729 | DG(29:1) | 0,000128 |
| 692 | SM(d35:0) | 0,000146 |  | 730 | PC(O-38:2) | 0,000127 |
| 693 | PC(45:11) | 0,000146 |  | 731 | PE(O-37:6) | 0,000127 |
| 694 | PE(34:3) | 0,000146 |  | 732 | DG(28:2) | 0,000127 |
| 695 | PC(40:3) | 0,000145 |  | 733 | PC(43:9) | 0,000126 |
| 696 | TG(39:0) | 0,000145 |  | 734 | LPC(O-20:1) | 0,000126 |
| 697 | PE(42:11) | 0,000145 |  | 735 | DG(41:4) | 0,000125 |
| 698 | TG(40:3) | 0,000144 |  | 736 | SM(t36:2) | 0,000125 |
| 699 | C1P(d34:1) | 0,000144 |  | 737 | DG(44:11) | 0,000125 |
| 700 | TG(O-52:3) | 0,000143 |  | 738 | TG(O-50:2) | 0,000124 |
| 701 | PE(32:1) | 0,000143 |  | 739 | Cer(d34:0) | 0,000123 |
| 702 | DG(41:1) | 0,000142 |  | 740 | LPC(O-26:1) | 0,000122 |
| 703 | PE(37:4) | 0,000142 |  | 741 | TG(57:5) | 0,000121 |
| 704 | DG(26:1) | 0,000141 |  | 742 | TG(63:2) | 0,000121 |
| 705 | PC(O-39:6) | 0,000139 |  | 743 | PC(O-39:5) | 0,00012 |
| 706 | Hex2Cer(d38:1) | 0,000139 |  | 744 | PE(O-35:3) | 0,00012 |
| 707 | TG(53:8) | 0,000138 |  | 745 | Cer(d39:0) | 0,000119 |
| 708 | LPC(24:1) | 0,000137 |  | 746 | SM(t33:1) | 0,000119 |
| 709 | DG(39:2) | 0,000137 |  | 747 | DG(39:7) | 0,000119 |
| 710 | PE(O-40:9) | 0,000137 |  | 748 | TG(44:5) | 0,000119 |
| 711 | SM(t40:2) | 0,000137 |  | 749 | TG(O-50:0) | 0,000118 |
| 712 | TG(46:6) | 0,000137 |  | 750 | TG(48:7) | 0,000118 |
| 713 | PI(35:1) | 0,000137 |  | 751 | Hex2Cer(d34:0) | 0,000117 |
| 714 | SM(d30:2) | 0,000137 |  | 752 | SM(d36:4) | 0,000117 |
| 715 | PI(35:2) | 0,000137 |  | 753 | SM(t41:1) | 0,000116 |
| 716 | PI(38:2) | 0,000135 |  | 754 | TG(62:15) | 0,000116 |
| 717 | TG(O-56:3) | 0,000134 |  | 755 | TG(O-58:4) | 0,000113 |
| 718 | DG(44:5) | 0,000133 |  | 756 | Hex2Cer(d36:1) | 0,000113 |
| 719 | PC(O-36:1) | 0,000133 |  | 757 | TG(O-54:3) | 0,000112 |
| 720 | DG(O-45:3) | 0,000133 |  | 758 | LPC(O-14:2) | 0,000112 |
| 721 | PC(O-44:9) | 0,000132 |  | 759 | DG(37:6) | 0,000112 |
| 722 | DG(30:3) | 0,000132 |  | 760 | SM(t38:1) | 0,000111 |

| 761 | TG(47:5) | 0,000111 |  | 799 | SM4(d42:2) | 9,26E-05 |
| --- | --- | --- | --- | --- | --- | --- |
| 762 | LPE(13:0) | 0,000111 |  | 800 | PE(O-38:8) | 9,26E-05 |
| 763 | SPH(18:0) | 0,00011 |  | 801 | SM(d33:0) | 9,25E-05 |
| 764 | TG(42:4) | 0,00011 |  | 802 | LPC(26:0) | 9,22E-05 |
| 765 | PE(39:6) | 0,00011 |  | 803 | LPE(18:3) | 9,18E-05 |
| 766 | DG(31:2) | 0,000109 |  | 804 | TG(62:8) | 9,18E-05 |
| 767 | SM4(t42:1) | 0,000109 |  | 805 | SM(t41:2) | 9,08E-05 |
| 768 | TG(62:7) | 0,000109 |  | 806 | DG(O-34:0) | 9,02E-05 |
| 769 | PC(30:2) | 0,000109 |  | 807 | DG(29:2) | 8,99E-05 |
| 770 | TG(51:7) | 0,000108 |  | 808 | TG(39:1) | 8,98E-05 |
| 771 | SM4(t40:1) | 0,000108 |  | 809 | PC(O-34:0) | 8,97E-05 |
| 772 | DG(O-36:1) | 0,000107 |  | 810 | LPE(16:1) | 8,89E-05 |
| 773 | PC(O-42:9) | 0,000107 |  | 811 | TG(41:2) | 8,75E-05 |
| 774 | DG(39:1) | 0,000106 |  | 812 | C1P(d50:1) | 8,74E-05 |
| 775 | TG(50:8) | 0,000105 |  | 813 | PE(O-46:8) | 8,65E-05 |
| 776 | TG(O-60:3) | 0,000104 |  | 814 | PE(38:2) | 8,64E-05 |
| 777 | DG(22:0) | 0,000104 |  | 815 | PC(O-39:7) | 8,64E-05 |
| 778 | PC(40:9) | 0,000104 |  | 816 | TG(64:1) | 8,6E-05 |
| 779 | TG(58:13) | 0,000104 |  | 817 | DG(O-36:2) | 8,55E-05 |
| 780 | TG(49:6) | 0,000102 |  | 818 | PE(O-42:9) | 8,53E-05 |
| 781 | DG(O-42:8) | 0,000101 |  | 819 | PE(O-38:2) | 8,51E-05 |
| 782 | TG(57:4) | 0,000101 |  | 820 | TG(34:1) | 8,51E-05 |
| 783 | PC(O-31:1) | 0,0001 |  | 821 | PC(O-44:3) | 8,46E-05 |
| 784 | TG(62:11) | 0,0001 |  | 822 | DG(32:4) | 8,44E-05 |
| 785 | DG(27:0) | 9,93E-05 |  | 823 | Cer(d46:0) | 8,41E-05 |
| 786 | PC(O-43:5) | 9,87E-05 |  | 824 | TG(63:3) | 8,38E-05 |
| 787 | PE(35:2) | 9,86E-05 |  | 825 | Cer(d35:1) | 8,37E-05 |
| 788 | HexCer(d31:2) | 9,86E-05 |  | 826 | TG(64:4) | 8,35E-05 |
| 789 | SM(d37:0) | 9,81E-05 |  | 827 | Cer(d43:3) | 8,34E-05 |
| 790 | TG(61:4) | 9,81E-05 |  | 828 | DG(44:7) | 8,19E-05 |
| 791 | SM(t40:0) | 9,78E-05 |  | 829 | DG(O-32:1) | 8,14E-05 |
| 792 | Cer(d38:2) | 9,75E-05 |  | 830 | PC(44:6) | 8,08E-05 |
| 793 | Cer(d26:1) | 9,71E-05 |  | 831 | TG(64:7) | 7,95E-05 |
| 794 | TG(O-60:4) | 9,68E-05 |  | 832 | PE(O-42:4) | 7,89E-05 |
| 795 | TG(57:10) | 9,66E-05 |  | 833 | SM(d42:7) | 7,88E-05 |
| 796 | PC(O-48:7) | 9,51E-05 |  | 834 | DG(41:2) | 7,82E-05 |
| 797 | DG(O-32:0) | 9,5E-05 |  | 835 | TG(37:0) | 7,82E-05 |
| 798 | TG(63:1) | 9,31E-05 |  | 836 | DG(O-35:1) | 7,81E-05 |

| 837 | LPC(O-17:1) | 7,73E-05 |  | 875 | PC(34:5) | 6,49E-05 |
| --- | --- | --- | --- | --- | --- | --- |
| 838 | PC(42:2) | 7,49E-05 |  | 876 | PC(O-41:4) | 6,49E-05 |
| 839 | DG(43:0) | 7,47E-05 |  | 877 | PC(O-39:4) | 6,46E-05 |
| 840 | DG(44:1) | 7,42E-05 |  | 878 | DG(44:2) | 6,41E-05 |
| 841 | SM4(t34:2) | 7,41E-05 |  | 879 | PC(44:9) | 6,4E-05 |
| 842 | TG(O-58:1) | 7,39E-05 |  | 880 | TG(67:6) | 6,39E-05 |
| 843 | PC(44:10) | 7,38E-05 |  | 881 | TG(62:10) | 6,38E-05 |
| 844 | DG(44:9) | 7,35E-05 |  | 882 | PE(O-37:4) | 6,36E-05 |
| 845 | Cer(d44:3) | 7,33E-05 |  | 883 | PC(O-46:10) | 6,32E-05 |
| 846 | TG(O-52:0) | 7,33E-05 |  | 884 | TG(O-54:4) | 6,31E-05 |
| 847 | TG(65:2) | 7,3E-05 |  | 885 | SM(d39:3) | 6,3E-05 |
| 848 | SM(d44:5) | 7,29E-05 |  | 886 | TG(45:4) | 6,26E-05 |
| 849 | PE(O-40:3) | 7,23E-05 |  | 887 | SM4(d40:1) | 6,26E-05 |
| 850 | PC(44:12) | 7,19E-05 |  | 888 | PE(O-41:7) | 6,26E-05 |
| 851 | PE(O-44:5) | 7,15E-05 |  | 889 | TG(36:2) | 6,25E-05 |
| 852 | LPE(O-20:1) | 7,14E-05 |  | 890 | PE(35:1) | 6,25E-05 |
| 853 | TG(38:3) | 7,14E-05 |  | 891 | SM(t33:2) | 6,24E-05 |
| 854 | PC(O-31:0) | 7,13E-05 |  | 892 | SM(t36:0) | 6,23E-05 |
| 855 | DG(O-33:5) | 7,13E-05 |  | 893 | HexCer(d44:2) | 6,22E-05 |
| 856 | PC(O-48:8) | 7,06E-05 |  | 894 | Cer(d46:1) | 6,17E-05 |
| 857 | TG(43:3) | 7,04E-05 |  | 895 | HexCer(d44:1) | 6,14E-05 |
| 858 | TG(66:3) | 7,04E-05 |  | 896 | DG(O-33:4) | 6,12E-05 |
| 859 | PE(40:8) | 7,01E-05 |  | 897 | TG(35:0) | 6,11E-05 |
| 860 | PC(41:7) | 7E-05 |  | 898 | PE(39:4) | 6,05E-05 |
| 861 | TG(66:2) | 6,89E-05 |  | 899 | PI(34:3) | 6,01E-05 |
| 862 | SM(d43:5) | 6,87E-05 |  | 900 | TG(O-60:5) | 6E-05 |
| 863 | TG(62:0) | 6,87E-05 |  | 901 | DG(O-33:1) | 5,96E-05 |
| 864 | Cer(d29:1) | 6,81E-05 |  | 902 | PC(O-35:4) | 5,96E-05 |
| 865 | PC(44:8) | 6,77E-05 |  | 903 | DG(O-34:2) | 5,87E-05 |
| 866 | TG(O-48:0) | 6,69E-05 |  | 904 | DG(43:1) | 5,86E-05 |
| 867 | PA(34:2) | 6,68E-05 |  | 905 | DG(43:7) | 5,81E-05 |
| 868 | SM(d38:4) | 6,67E-05 |  | 906 | HexCer(d40:0) | 5,78E-05 |
| 869 | PC(O-43:4) | 6,66E-05 |  | 907 | PI(39:4) | 5,73E-05 |
| 870 | SM(t35:1) | 6,64E-05 |  | 908 | LPC(26:1) | 5,7E-05 |
| 871 | PC(40:2) | 6,63E-05 |  | 909 | PE(O-44:9) | 5,7E-05 |
| 872 | PC(42:11) | 6,61E-05 |  | 910 | PC(44:7) | 5,62E-05 |
| 873 | SM4(t41:1) | 6,59E-05 |  | 911 | PC(O-40:1) | 5,57E-05 |
| 874 | HexCer(d34:2) | 6,57E-05 |  | 912 | TG(61:0) | 5,52E-05 |

| 913 | C1P(d44:1) | 5,5E-05 |  | 951 | TG(42:6) | 4,89E-05 |
| --- | --- | --- | --- | --- | --- | --- |
| 914 | PE(O-35:5) | 5,49E-05 |  | 952 | Cer(d46:2) | 4,87E-05 |
| 915 | TG(65:3) | 5,47E-05 |  | 953 | PE(O-36:1) | 4,86E-05 |
| 916 | PE(O-37:7) | 5,46E-05 |  | 954 | PE(O-34:4) | 4,82E-05 |
| 917 | LPC(16:2) | 5,43E-05 |  | 955 | SM(t39:1) | 4,78E-05 |
| 918 | SM(t32:2) | 5,41E-05 |  | 956 | PI(37:3) | 4,77E-05 |
| 919 | TG(O-60:2) | 5,41E-05 |  | 957 | TG(49:7) | 4,77E-05 |
| 920 | LPC(O-26:2) | 5,39E-05 |  | 958 | LPC(22:3) | 4,76E-05 |
| 921 | Cer(d45:0) | 5,39E-05 |  | 959 | LPC(24:4) | 4,76E-05 |
| 922 | PC(O-41:5) | 5,38E-05 |  | 960 | TG(40:4) | 4,75E-05 |
| 923 | PC(46:5) | 5,33E-05 |  | 961 | TG(64:5) | 4,71E-05 |
| 924 | TG(56:12) | 5,33E-05 |  | 962 | Cer(d48:0) | 4,71E-05 |
| 925 | PE(38:7) | 5,3E-05 |  | 963 | PC(O-28:0) | 4,68E-05 |
| 926 | TG(O-58:5) | 5,3E-05 |  | 964 | SM4(t34:0) | 4,65E-05 |
| 927 | TG(42:5) | 5,24E-05 |  | 965 | SM4(d42:3) | 4,59E-05 |
| 928 | TG(62:9) | 5,23E-05 |  | 966 | PC(O-42:1) | 4,59E-05 |
| 929 | TG(37:1) | 5,22E-05 |  | 967 | PE(42:7) | 4,56E-05 |
| 930 | SM(d29:1) | 5,21E-05 |  | 968 | C1P(d42:2) | 4,55E-05 |
| 931 | SM4(d34:2) | 5,2E-05 |  | 969 | TG(64:16) | 4,55E-05 |
| 932 | PC(42:3) | 5,2E-05 |  | 970 | TG(44:6) | 4,52E-05 |
| 933 | LPC(23:1) | 5,17E-05 |  | 971 | PC(44:11) | 4,51E-05 |
| 934 | PC(28:1) | 5,15E-05 |  | 972 | TG(47:6) | 4,49E-05 |
| 935 | DG(42:4) | 5,15E-05 |  | 973 | PE(42:10) | 4,49E-05 |
| 936 | DG(O-42:6) | 5,14E-05 |  | 974 | LPE(O-24:2) | 4,48E-05 |
| 937 | PI(34:0) | 5,13E-05 |  | 975 | PE(38:1) | 4,48E-05 |
| 938 | TG(O-56:6) | 5,12E-05 |  | 976 | TG(54:11) | 4,47E-05 |
| 939 | DG(O-38:1) | 5,07E-05 |  | 977 | Cer(d47:0) | 4,44E-05 |
| 940 | PC(39:3) | 5,04E-05 |  | 978 | LPE(O-22:1) | 4,44E-05 |
| 941 | PI(33:1) | 5,02E-05 |  | 979 | TG(O-56:4) | 4,44E-05 |
| 942 | PC(46:6) | 5E-05 |  | 980 | PA(34:1) | 4,43E-05 |
| 943 | SM(t42:0) | 5E-05 |  | 981 | SM(d30:0) | 4,42E-05 |
| 944 | LPC(O-18:3) | 4,98E-05 |  | 982 | TG(O-53:1) | 4,39E-05 |
| 945 | DG(O-35:2) | 4,97E-05 |  | 983 | Cer(d36:2) | 4,39E-05 |
| 946 | PC(36:0) | 4,96E-05 |  | 984 | PC(O-37:6) | 4,38E-05 |
| 947 | DG(O-40:1) | 4,96E-05 |  | 985 | PC(37:1) | 4,34E-05 |
| 948 | DG(O-47:4) | 4,95E-05 |  | 986 | PC(41:5) | 4,34E-05 |
| 949 | TG(59:11) | 4,92E-05 |  | 987 | TG(59:5) | 4,34E-05 |
| 950 | PC(33:3) | 4,91E-05 |  | 988 | SM(t38:2) | 4,33E-05 |

| 989 | SM(d37:3) | 4,32E-05 |  | 1027 | PC(O-46:4) | 3,77E-05 |
| --- | --- | --- | --- | --- | --- | --- |
| 990 | SM4(d42:1) | 4,32E-05 |  | 1028 | DG(41:3) | 3,77E-05 |
| 991 | TG(55:10) | 4,32E-05 |  | 1029 | DG(32:5) | 3,76E-05 |
| 992 | PI(40:7) | 4,28E-05 |  | 1030 | LPC(19:2) | 3,75E-05 |
| 993 | TG(65:1) | 4,26E-05 |  | 1031 | PA(36:2) | 3,74E-05 |
| 994 | PC(O-38:1) | 4,25E-05 |  | 1032 | SM(d44:6) | 3,71E-05 |
| 995 | TG(O-60:6) | 4,24E-05 |  | 1033 | DG(O-36:0) | 3,71E-05 |
| 996 | LPC(O-20:2) | 4,21E-05 |  | 1034 | LPA(20:3) | 3,7E-05 |
| 997 | LPE(O-24:1) | 4,19E-05 |  | 1035 | PC(46:4) | 3,69E-05 |
| 998 | DG(O-31:1) | 4,18E-05 |  | 1036 | PE(39:5) | 3,68E-05 |
| 999 | TG(63:4) | 4,18E-05 |  | 1037 | TG(O-54:0) | 3,67E-05 |
| 1000 | PE(42:6) | 4,17E-05 |  | 1038 | DG(O-47:3) | 3,66E-05 |
| 1001 | SM(d41:4) | 4,16E-05 |  | 1039 | TG(O-52:4) | 3,66E-05 |
| 1002 | SM4(t40:2) | 4,15E-05 |  | 1040 | Cer(d48:1) | 3,64E-05 |
| 1003 | TG(66:4) | 4,13E-05 |  | 1041 | LPE(20:1) | 3,63E-05 |
| 1004 | PA(38:4) | 4,11E-05 |  | 1042 | DG(O-37:2) | 3,63E-05 |
| 1005 | Cer(d42:4) | 4,09E-05 |  | 1043 | PC(O-45:5) | 3,62E-05 |
| 1006 | DG(27:1) | 4,06E-05 |  | 1044 | SM(t30:2) | 3,62E-05 |
| 1007 | SM4(d35:0) | 4,04E-05 |  | 1045 | PE(26:0) | 3,62E-05 |
| 1008 | PE(O-42:3) | 4,01E-05 |  | 1046 | SM(d35:3) | 3,61E-05 |
| 1009 | PE(O-35:2) | 3,97E-05 |  | 1047 | LPA(16:1) | 3,6E-05 |
| 1010 | SPH(20:0) | 3,97E-05 |  | 1048 | PC(O-43:6) | 3,59E-05 |
| 1011 | Cer(d48:2) | 3,95E-05 |  | 1049 | LPA(22:6) | 3,57E-05 |
| 1012 | TG(O-48:1) | 3,95E-05 |  | 1050 | C1P(d43:1) | 3,56E-05 |
| 1013 | PI(36:5) | 3,94E-05 |  | 1051 | DG(44:0) | 3,53E-05 |
| 1014 | PC(O-42:10) | 3,94E-05 |  | 1052 | DG(43:2) | 3,49E-05 |
| 1015 | Cer(d33:1) | 3,93E-05 |  | 1053 | C1P(d41:1) | 3,46E-05 |
| 1016 | TG(63:8) | 3,9E-05 |  | 1054 | TG(64:17) | 3,46E-05 |
| 1017 | TG(59:12) | 3,87E-05 |  | 1055 | TG(46:7) | 3,43E-05 |
| 1018 | SM4(d40:2) | 3,85E-05 |  | 1056 | DG(O-46:3) | 3,39E-05 |
| 1019 | PC(39:7) | 3,85E-05 |  | 1057 | PE(O-32:1) | 3,38E-05 |
| 1020 | PC(O-48:6) | 3,84E-05 |  | 1058 | PC(O-33:2) | 3,38E-05 |
| 1021 | TG(O-56:7) | 3,84E-05 |  | 1059 | PC(O-40:9) | 3,34E-05 |
| 1022 | TG(33:0) | 3,83E-05 |  | 1060 | PC(46:7) | 3,34E-05 |
| 1023 | LPC(23:0) | 3,81E-05 |  | 1061 | SM4(t42:3) | 3,33E-05 |
| 1024 | SM(t41:0) | 3,79E-05 |  | 1062 | Cer(d47:1) | 3,32E-05 |
| 1025 | TG(66:1) | 3,79E-05 |  | 1063 | PE(O-46:7) | 3,32E-05 |
| 1026 | TG(57:11) | 3,77E-05 |  | 1064 | DG(44:6) | 3,3E-05 |

| 1065 | LPC(22:2) | 3,3E-05 |  | 1103 | PE(O-40:2) | 2,79E-05 |
| --- | --- | --- | --- | --- | --- | --- |
| 1066 | Hex2Cer(d44:2) | 3,3E-05 |  | 1104 | TG(O-58:6) | 2,77E-05 |
| 1067 | PE(O-32:2) | 3,29E-05 |  | 1105 | TG(51:8) | 2,76E-05 |
| 1068 | PA(36:4) | 3,28E-05 |  | 1106 | LPE(O-17:1) | 2,75E-05 |
| 1069 | TG(52:10) | 3,27E-05 |  | 1107 | DG(O-42:2) | 2,75E-05 |
| 1070 | Cer(d28:1) | 3,27E-05 |  | 1108 | LPC(O-14:0) | 2,74E-05 |
| 1071 | TG(64:8) | 3,24E-05 |  | 1109 | DG(O-39:1) | 2,74E-05 |
| 1072 | TG(64:0) | 3,23E-05 |  | 1110 | SM4(t41:2) | 2,73E-05 |
| 1073 | LPC(14:1) | 3,22E-05 |  | 1111 | PE(O-41:8) | 2,72E-05 |
| 1074 | TG(48:8) | 3,18E-05 |  | 1112 | SM4(d41:1) | 2,72E-05 |
| 1075 | TG(O-54:6) | 3,18E-05 |  | 1113 | PE(32:0) | 2,72E-05 |
| 1076 | PI(30:0) | 3,16E-05 |  | 1114 | Cer(d49:1) | 2,71E-05 |
| 1077 | PC(O-45:6) | 3,14E-05 |  | 1115 | PE(O-46:9) | 2,71E-05 |
| 1078 | TG(53:9) | 3,12E-05 |  | 1116 | SM(d31:2) | 2,7E-05 |
| 1079 | Cer(d50:0) | 3,12E-05 |  | 1117 | PC(O-35:2) | 2,68E-05 |
| 1080 | TG(O-51:1) | 3,1E-05 |  | 1118 | TG(50:9) | 2,67E-05 |
| 1081 | Cer(d50:1) | 3,1E-05 |  | 1119 | TG(59:8) | 2,67E-05 |
| 1082 | Cer(d41:3) | 3,09E-05 |  | 1120 | DG(O-43:3) | 2,64E-05 |
| 1083 | PC(44:3) | 3,07E-05 |  | 1121 | TG(O-54:5) | 2,63E-05 |
| 1084 | HexCer(d42:0) | 3,06E-05 |  | 1122 | PC(35:0) | 2,62E-05 |
| 1085 | PE(41:6) | 3,06E-05 |  | 1123 | DG(O-38:2) | 2,62E-05 |
| 1086 | TG(63:0) | 3,04E-05 |  | 1124 | TG(O-53:2) | 2,59E-05 |
| 1087 | TG(61:5) | 3,04E-05 |  | 1125 | TG(O-60:7) | 2,58E-05 |
| 1088 | DG(O-40:2) | 3,03E-05 |  | 1126 | TG(O-51:0) | 2,58E-05 |
| 1089 | TG(65:4) | 3,01E-05 |  | 1127 | TG(O-56:5) | 2,55E-05 |
| 1090 | Cer(d49:0) | 2,99E-05 |  | 1128 | TG(45:5) | 2,55E-05 |
| 1091 | LPE(O-16:0) | 2,98E-05 |  | 1129 | DG(O-33:2) | 2,53E-05 |
| 1092 | TG(39:2) | 2,97E-05 |  | 1130 | SM(t38:0) | 2,5E-05 |
| 1093 | TG(66:8) | 2,96E-05 |  | 1131 | PC(O-48:9) | 2,49E-05 |
| 1094 | TG(59:7) | 2,96E-05 |  | 1132 | PC(O-44:10) | 2,49E-05 |
| 1095 | TG(O-62:7) | 2,95E-05 |  | 1133 | TG(67:2) | 2,46E-05 |
| 1096 | PE(37:2) | 2,93E-05 |  | 1134 | TG(O-55:1) | 2,46E-05 |
| 1097 | C1P(d40:1) | 2,93E-05 |  | 1135 | PC(O-44:2) | 2,46E-05 |
| 1098 | TG(59:10) | 2,89E-05 |  | 1136 | DG(O-36:3) | 2,46E-05 |
| 1099 | PS(34:1) | 2,87E-05 |  | 1137 | DG(O-31:0) | 2,46E-05 |
| 1100 | PC(O-48:10) | 2,84E-05 |  | 1138 | LPC(15:1) | 2,46E-05 |
| 1101 | LPE(O-20:2) | 2,84E-05 |  | 1139 | TG(O-56:0) | 2,45E-05 |
| 1102 | PC(24:0) | 2,82E-05 |  | 1140 | TG(O-55:2) | 2,44E-05 |

| 1141 | LPE(17:0) | 2,44E-05 |  | 1179 | PC(O-33:1) | 2,13E-05 |
| --- | --- | --- | --- | --- | --- | --- |
| 1142 | LPC(25:0) | 2,43E-05 |  | 1180 | PE(42:8) | 2,12E-05 |
| 1143 | Cer(d34:2) | 2,42E-05 |  | 1181 | TG(59:6) | 2,12E-05 |
| 1144 | DG(O-45:2) | 2,42E-05 |  | 1182 | PE(O-41:6) | 2,11E-05 |
| 1145 | PC(O-41:6) | 2,42E-05 |  | 1183 | DG(O-33:0) | 2,1E-05 |
| 1146 | DG(O-44:6) | 2,4E-05 |  | 1184 | PC(40:1) | 2,09E-05 |
| 1147 | TG(65:0) | 2,37E-05 |  | 1185 | DG(O-42:5) | 2,07E-05 |
| 1148 | DG(46:7) | 2,37E-05 |  | 1186 | PE(O-36:7) | 2,07E-05 |
| 1149 | PA(36:3) | 2,34E-05 |  | 1187 | DG(44:3) | 2,06E-05 |
| 1150 | PE(40:3) | 2,33E-05 |  | 1188 | TG(O-58:7) | 2,06E-05 |
| 1151 | LPC(O-23:0) | 2,33E-05 |  | 1189 | LPC(26:3) | 2,05E-05 |
| 1152 | SM(d44:7) | 2,33E-05 |  | 1190 | TG(63:6) | 2,05E-05 |
| 1153 | TG(63:5) | 2,32E-05 |  | 1191 | TG(O-57:1) | 2,05E-05 |
| 1154 | SM(d43:0) | 2,31E-05 |  | 1192 | PC(O-45:7) | 2,04E-05 |
| 1155 | PC(27:0) | 2,3E-05 |  | 1193 | LPE(20:2) | 2,04E-05 |
| 1156 | PS(40:7) | 2,29E-05 |  | 1194 | TG(66:0) | 2,04E-05 |
| 1157 | PE(O-41:5) | 2,29E-05 |  | 1195 | PE(34:0) | 2,04E-05 |
| 1158 | TG(O-51:2) | 2,27E-05 |  | 1196 | TG(59:9) | 2,03E-05 |
| 1159 | PC(44:2) | 2,27E-05 |  | 1197 | LPE(24:6) | 2,02E-05 |
| 1160 | TG(68:2) | 2,27E-05 |  | 1198 | TG(68:4) | 2,02E-05 |
| 1161 | TG(68:3) | 2,26E-05 |  | 1199 | SM(t43:1) | 2,01E-05 |
| 1162 | PE(42:5) | 2,25E-05 |  | 1200 | Cer(d37:0) | 2,01E-05 |
| 1163 | DG(41:6) | 2,24E-05 |  | 1201 | SM(t32:0) | 1,99E-05 |
| 1164 | TG(66:5) | 2,24E-05 |  | 1202 | PC(O-35:5) | 1,98E-05 |
| 1165 | PC(O-37:3) | 2,24E-05 |  | 1203 | PC(43:4) | 1,98E-05 |
| 1166 | PC(35:5) | 2,23E-05 |  | 1204 | TG(66:6) | 1,97E-05 |
| 1167 | Cer(d39:2) | 2,22E-05 |  | 1205 | TG(O-60:1) | 1,96E-05 |
| 1168 | TG(67:3) | 2,22E-05 |  | 1206 | TG(61:6) | 1,96E-05 |
| 1169 | LPC(O-24:3) | 2,22E-05 |  | 1207 | LPE(O-22:2) | 1,94E-05 |
| 1170 | DG(O-39:2) | 2,21E-05 |  | 1208 | SM4(d32:1) | 1,94E-05 |
| 1171 | DG(O-37:1) | 2,19E-05 |  | 1209 | LPA(18:3) | 1,93E-05 |
| 1172 | SM(t37:1) | 2,18E-05 |  | 1210 | PE(33:1) | 1,89E-05 |
| 1173 | TG(O-62:6) | 2,17E-05 |  | 1211 | DG(O-31:2) | 1,88E-05 |
| 1174 | Cer(d45:2) | 2,17E-05 |  | 1212 | SM(d43:4) | 1,87E-05 |
| 1175 | TG(O-57:2) | 2,15E-05 |  | 1213 | SM(t36:3) | 1,85E-05 |
| 1176 | Hex2Cer(d34:2) | 2,15E-05 |  | 1214 | LPC(13:0) | 1,83E-05 |
| 1177 | TG(O-50:3) | 2,14E-05 |  | 1215 | PA(26:0) | 1,82E-05 |
| 1178 | PC(O-41:7) | 2,14E-05 |  | 1216 | DG(O-43:2) | 1,82E-05 |

| 1217 | PE(37:3) | 1,81E-05 |  | 1255 | Cer(d46:3) | 1,55E-05 |
| --- | --- | --- | --- | --- | --- | --- |
| 1218 | PC(41:4) | 1,8E-05 |  | 1256 | PC(46:8) | 1,54E-05 |
| 1219 | TG(67:4) | 1,8E-05 |  | 1257 | DG(O-44:2) | 1,54E-05 |
| 1220 | TG(35:1) | 1,79E-05 |  | 1258 | PE(O-33:2) | 1,53E-05 |
| 1221 | HexCer(d30:2) | 1,79E-05 |  | 1259 | TG(32:1) | 1,52E-05 |
| 1222 | LPC(28:0) | 1,79E-05 |  | 1260 | SM(d32:3) | 1,52E-05 |
| 1223 | PE(O-35:4) | 1,77E-05 |  | 1261 | Cer(d31:1) | 1,52E-05 |
| 1224 | SM4(t32:1) | 1,76E-05 |  | 1262 | TG(O-48:2) | 1,51E-05 |
| 1225 | DG(41:7) | 1,76E-05 |  | 1263 | DG(O-42:1) | 1,51E-05 |
| 1226 | TG(64:15) | 1,75E-05 |  | 1264 | TG(68:5) | 1,5E-05 |
| 1227 | TG(41:3) | 1,74E-05 |  | 1265 | Cer(d32:2) | 1,49E-05 |
| 1228 | SM(t30:1) | 1,72E-05 |  | 1266 | PE(44:11) | 1,48E-05 |
| 1229 | TG(67:1) | 1,72E-05 |  | 1267 | TG(61:7) | 1,48E-05 |
| 1230 | SM4(d36:1) | 1,72E-05 |  | 1268 | LPE(24:5) | 1,47E-05 |
| 1231 | PC(42:1) | 1,71E-05 |  | 1269 | LPC(17:2) | 1,47E-05 |
| 1232 | PE(O-39:8) | 1,71E-05 |  | 1270 | SM4(d34:0) | 1,47E-05 |
| 1233 | PE(O-34:5) | 1,71E-05 |  | 1271 | PA(36:1) | 1,46E-05 |
| 1234 | SM4(t38:1) | 1,7E-05 |  | 1272 | SM4(d37:1) | 1,46E-05 |
| 1235 | DG(O-44:7) | 1,7E-05 |  | 1273 | DG(O-44:3) | 1,46E-05 |
| 1236 | PC(O-33:3) | 1,7E-05 |  | 1274 | PA(38:6) | 1,46E-05 |
| 1237 | PE(O-37:3) | 1,69E-05 |  | 1275 | DG(30:4) | 1,45E-05 |
| 1238 | DG(O-45:4) | 1,68E-05 |  | 1276 | PC(31:2) | 1,45E-05 |
| 1239 | DG(O-38:0) | 1,68E-05 |  | 1277 | SM(d28:2) | 1,45E-05 |
| 1240 | LPC(O-15:1) | 1,67E-05 |  | 1278 | TG(O-59:2) | 1,45E-05 |
| 1241 | SM4(d41:2) | 1,67E-05 |  | 1279 | SM(d45:2) | 1,43E-05 |
| 1242 | LPC(29:0) | 1,65E-05 |  | 1280 | PC(45:10) | 1,42E-05 |
| 1243 | LPE(O-18:0) | 1,64E-05 |  | 1281 | TG(63:7) | 1,41E-05 |
| 1244 | PC(32:4) | 1,64E-05 |  | 1282 | PC(O-38:8) | 1,41E-05 |
| 1245 | Hex2Cer(d28:1) | 1,61E-05 |  | 1283 | TG(68:1) | 1,4E-05 |
| 1246 | PC(O-36:0) | 1,61E-05 |  | 1284 | TG(O-53:0) | 1,4E-05 |
| 1247 | PS(42:6) | 1,6E-05 |  | 1285 | DG(46:2) | 1,4E-05 |
| 1248 | SM(t43:2) | 1,58E-05 |  | 1286 | PI(30:1) | 1,39E-05 |
| 1249 | DG(O-47:5) | 1,58E-05 |  | 1287 | LPE(24:0) | 1,39E-05 |
| 1250 | PE(O-42:10) | 1,57E-05 |  | 1288 | TG(65:5) | 1,39E-05 |
| 1251 | PC(43:8) | 1,57E-05 |  | 1289 | LPC(26:2) | 1,38E-05 |
| 1252 | DG(O-42:3) | 1,57E-05 |  | 1290 | TG(O-62:5) | 1,38E-05 |
| 1253 | TG(O-62:8) | 1,55E-05 |  | 1291 | TG(O-59:1) | 1,34E-05 |
| 1254 | TG(43:4) | 1,55E-05 |  | 1292 | SM(t31:1) | 1,34E-05 |

| 1293 | SM(t31:2) | 1,34E-05 |  | 1331 | TG(66:9) | 1,1E-05 |
| --- | --- | --- | --- | --- | --- | --- |
| 1294 | SM(d28:0) | 1,33E-05 |  | 1332 | TG(64:14) | 1,09E-05 |
| 1295 | TG(O-57:3) | 1,33E-05 |  | 1333 | DG(46:6) | 1,09E-05 |
| 1296 | TG(61:13) | 1,32E-05 |  | 1334 | LPC(21:5) | 1,08E-05 |
| 1297 | DG(O-41:2) | 1,32E-05 |  | 1335 | TG(37:2) | 1,08E-05 |
| 1298 | LPE(O-24:0) | 1,32E-05 |  | 1336 | LPC(10:0) | 1,07E-05 |
| 1299 | TG(66:18) | 1,31E-05 |  | 1337 | TG(44:7) | 1,06E-05 |
| 1300 | PE(42:9) | 1,3E-05 |  | 1338 | HexCer(d44:3) | 1,05E-05 |
| 1301 | PC(O-43:7) | 1,3E-05 |  | 1339 | PS(26:0) | 1,05E-05 |
| 1302 | SM(t35:2) | 1,3E-05 |  | 1340 | PE(O-46:6) | 1,05E-05 |
| 1303 | PE(O-33:3) | 1,29E-05 |  | 1341 | SM4(d33:1) | 1,05E-05 |
| 1304 | PC(43:10) | 1,28E-05 |  | 1342 | SM(d26:1) | 1,04E-05 |
| 1305 | TG(O-58:0) | 1,28E-05 |  | 1343 | HexCer(d38:0) | 1,03E-05 |
| 1306 | SM4(t36:1) | 1,27E-05 |  | 1344 | TG(38:4) | 1,03E-05 |
| 1307 | TG(54:12) | 1,27E-05 |  | 1345 | TG(O-64:8) | 1,02E-05 |
| 1308 | LPE(O-18:3) | 1,26E-05 |  | 1346 | LPE(22:7) | 1,02E-05 |
| 1309 | PE(36:6) | 1,25E-05 |  | 1347 | TG(68:6) | 1,01E-05 |
| 1310 | PE(32:2) | 1,23E-05 |  | 1348 | LPC(O-26:0) | 1,01E-05 |
| 1311 | PI(39:5) | 1,23E-05 |  | 1349 | DG(O-40:3) | 9,99E-06 |
| 1312 | LPC(19:3) | 1,23E-05 |  | 1350 | PE(30:0) | 9,97E-06 |
| 1313 | TG(67:0) | 1,23E-05 |  | 1351 | PI(32:2) | 9,89E-06 |
| 1314 | PC(O-48:5) | 1,21E-05 |  | 1352 | TG(O-62:3) | 9,89E-06 |
| 1315 | SM4(t39:1) | 1,21E-05 |  | 1353 | SM(d45:3) | 9,88E-06 |
| 1316 | DG(O-35:0) | 1,21E-05 |  | 1354 | LPA(17:0) | 9,88E-06 |
| 1317 | TG(61:12) | 1,19E-05 |  | 1355 | PC(O-41:3) | 9,83E-06 |
| 1318 | PE(34:4) | 1,19E-05 |  | 1356 | LPC(O-20:3) | 9,76E-06 |
| 1319 | PC(43:5) | 1,18E-05 |  | 1357 | LPC(21:0) | 9,7E-06 |
| 1320 | TG(O-62:4) | 1,16E-05 |  | 1358 | PE(O-34:0) | 9,64E-06 |
| 1321 | PC(O-33:0) | 1,16E-05 |  | 1359 | PE(O-44:4) | 9,55E-06 |
| 1322 | LPC(24:2) | 1,15E-05 |  | 1360 | DG(45:6) | 9,49E-06 |
| 1323 | PE(O-42:2) | 1,15E-05 |  | 1361 | TG(O-55:0) | 9,25E-06 |
| 1324 | PC(O-28:1) | 1,12E-05 |  | 1362 | TG(47:7) | 9,23E-06 |
| 1325 | TG(32:2) | 1,12E-05 |  | 1363 | C1P(d45:1) | 9,13E-06 |
| 1326 | TG(O-59:3) | 1,11E-05 |  | 1364 | SM4(t40:0) | 9,13E-06 |
| 1327 | TG(O-55:3) | 1,11E-05 |  | 1365 | PC(45:4) | 8,92E-06 |
| 1328 | LPC(O-22:3) | 1,11E-05 |  | 1366 | TG(68:7) | 8,89E-06 |
| 1329 | PI(31:0) | 1,1E-05 |  | 1367 | PE(42:4) | 8,87E-06 |
| 1330 | TG(40:5) | 1,1E-05 |  | 1368 | HexCer(d30:0) | 8,85E-06 |

| 1369 | TG(67:5) | 8,84E-06 |  | 1407 | LPC(O-24:4) | 7,21E-06 |
| --- | --- | --- | --- | --- | --- | --- |
| 1370 | PC(29:1) | 8,76E-06 |  | 1408 | TG(65:6) | 7,21E-06 |
| 1371 | TG(O-49:0) | 8,67E-06 |  | 1409 | TG(68:8) | 7,17E-06 |
| 1372 | TG(49:8) | 8,66E-06 |  | 1410 | TG(O-61:1) | 7,07E-06 |
| 1373 | LPC(O-17:0) | 8,65E-06 |  | 1411 | PE(33:2) | 7E-06 |
| 1374 | TG(55:11) | 8,61E-06 |  | 1412 | SM(d45:1) | 6,95E-06 |
| 1375 | LPC(21:1) | 8,48E-06 |  | 1413 | PE(O-32:0) | 6,94E-06 |
| 1376 | PC(O-32:3) | 8,47E-06 |  | 1414 | TG(O-54:7) | 6,93E-06 |
| 1377 | PE(O-43:7) | 8,43E-06 |  | 1415 | TG(69:3) | 6,88E-06 |
| 1378 | PC(O-46:11) | 8,41E-06 |  | 1416 | TG(70:4) | 6,87E-06 |
| 1379 | PA(38:5) | 8,38E-06 |  | 1417 | PC(46:9) | 6,8E-06 |
| 1380 | TG(O-64:7) | 8,36E-06 |  | 1418 | TG(33:1) | 6,69E-06 |
| 1381 | Hex2Cer(d44:1) | 8,24E-06 |  | 1419 | LPC(O-25:1) | 6,69E-06 |
| 1382 | TG(70:2) | 8,24E-06 |  | 1420 | TG(O-57:0) | 6,68E-06 |
| 1383 | PE(O-43:6) | 8,15E-06 |  | 1421 | TG(65:7) | 6,63E-06 |
| 1384 | PE(37:1) | 8,07E-06 |  | 1422 | SM4(t42:0) | 6,62E-06 |
| 1385 | SM4(t35:1) | 8,01E-06 |  | 1423 | SM4(d36:2) | 6,62E-06 |
| 1386 | TG(68:0) | 7,99E-06 |  | 1424 | PC(46:10) | 6,58E-06 |
| 1387 | LPC(24:3) | 7,98E-06 |  | 1425 | SM(t39:0) | 6,57E-06 |
| 1388 | SM(t37:2) | 7,97E-06 |  | 1426 | SM(t33:0) | 6,56E-06 |
| 1389 | SM4(t33:1) | 7,94E-06 |  | 1427 | TG(O-62:1) | 6,53E-06 |
| 1390 | TG(69:2) | 7,92E-06 |  | 1428 | SM(d26:2) | 6,47E-06 |
| 1391 | TG(O-46:1) | 7,87E-06 |  | 1429 | LPE(20:0) | 6,46E-06 |
| 1392 | SM4(t36:2) | 7,78E-06 |  | 1430 | TG(O-58:8) | 6,46E-06 |
| 1393 | Cer(d35:0) | 7,77E-06 |  | 1431 | TG(69:4) | 6,41E-06 |
| 1394 | TG(51:9) | 7,77E-06 |  | 1432 | TG(36:3) | 6,39E-06 |
| 1395 | PE(37:6) | 7,75E-06 |  | 1433 | TG(O-64:9) | 6,33E-06 |
| 1396 | SM4(d38:2) | 7,74E-06 |  | 1434 | SM(t35:0) | 6,27E-06 |
| 1397 | TG(O-53:3) | 7,74E-06 |  | 1435 | SM4(d38:1) | 6,25E-06 |
| 1398 | TG(O-62:2) | 7,7E-06 |  | 1436 | LPC(O-25:0) | 6,25E-06 |
| 1399 | PC(O-30:2) | 7,64E-06 |  | 1437 | PC(O-45:4) | 6,19E-06 |
| 1400 | TG(70:3) | 7,58E-06 |  | 1438 | PC(O-41:2) | 6,15E-06 |
| 1401 | LPC(26:4) | 7,51E-06 |  | 1439 | PC(48:6) | 6,14E-06 |
| 1402 | TG(48:9) | 7,41E-06 |  | 1440 | Cer(d45:3) | 6,1E-06 |
| 1403 | LPE(O-20:3) | 7,35E-06 |  | 1441 | TG(34:2) | 6,08E-06 |
| 1404 | TG(O-56:8) | 7,34E-06 |  | 1442 | SM(d45:4) | 6,06E-06 |
| 1405 | PE(O-43:5) | 7,24E-06 |  | 1443 | TG(69:1) | 6,05E-06 |
| 1406 | LPE(12:0) | 7,22E-06 |  | 1444 | DG(O-40:4) | 5,98E-06 |

| 1445 | PC(46:2) | 5,97E-06 |  | 1483 | PC(45:5) | 4,64E-06 |
| --- | --- | --- | --- | --- | --- | --- |
| 1446 | PE(O-30:1) | 5,93E-06 |  | 1484 | PC(44:1) | 4,64E-06 |
| 1447 | LPC(25:1) | 5,93E-06 |  | 1485 | LPC(30:0) | 4,57E-06 |
| 1448 | LPE(22:0) | 5,89E-06 |  | 1486 | TG(43:5) | 4,5E-06 |
| 1449 | TG(70:5) | 5,87E-06 |  | 1487 | PE(O-33:1) | 4,46E-06 |
| 1450 | DG(43:6) | 5,86E-06 |  | 1488 | LPC(O-23:1) | 4,46E-06 |
| 1451 | TG(53:10) | 5,86E-06 |  | 1489 | SM4(t41:0) | 4,38E-06 |
| 1452 | TG(O-61:2) | 5,84E-06 |  | 1490 | PC(O-31:2) | 4,36E-06 |
| 1453 | PC(O-47:7) | 5,83E-06 |  | 1491 | PC(O-45:8) | 4,34E-06 |
| 1454 | SM(d46:2) | 5,82E-06 |  | 1492 | SM(d27:1) | 4,31E-06 |
| 1455 | LPA(15:0) | 5,78E-06 |  | 1493 | SM4(t43:2) | 4,28E-06 |
| 1456 | PC(O-29:1) | 5,77E-06 |  | 1494 | TG(69:0) | 4,26E-06 |
| 1457 | PE(37:5) | 5,62E-06 |  | 1495 | LPC(O-21:0) | 4,25E-06 |
| 1458 | PE(35:3) | 5,59E-06 |  | 1496 | PC(O-43:3) | 4,24E-06 |
| 1459 | TG(52:11) | 5,57E-06 |  | 1497 | PC(48:5) | 4,23E-06 |
| 1460 | PE(O-31:1) | 5,49E-06 |  | 1498 | LPC(28:3) | 4,21E-06 |
| 1461 | PE(O-32:3) | 5,4E-06 |  | 1499 | TG(O-64:6) | 4,19E-06 |
| 1462 | TG(O-59:4) | 5,34E-06 |  | 1500 | PA(40:6) | 4,12E-06 |
| 1463 | TG(69:5) | 5,21E-06 |  | 1501 | LPC(28:1) | 4,11E-06 |
| 1464 | PE(O-35:6) | 5,19E-06 |  | 1502 | LPC(23:6) | 4,09E-06 |
| 1465 | TG(O-60:0) | 5,14E-06 |  | 1503 | PC(28:2) | 4,05E-06 |
| 1466 | TG(46:8) | 5,14E-06 |  | 1504 | TG(50:10) | 3,95E-06 |
| 1467 | PE(O-38:1) | 5,09E-06 |  | 1505 | SM4(t38:2) | 3,91E-06 |
| 1468 | TG(O-59:0) | 5,08E-06 |  | 1506 | LPE(O-19:1) | 3,69E-06 |
| 1469 | HexCer(d32:2) | 5,08E-06 |  | 1507 | TG(40:6) | 3,68E-06 |
| 1470 | TG(O-57:4) | 5,06E-06 |  | 1508 | Cer(d47:2) | 3,61E-06 |
| 1471 | DG(32:6) | 5,03E-06 |  | 1509 | LPC(28:4) | 3,6E-06 |
| 1472 | PC(48:7) | 4,96E-06 |  | 1510 | PC(41:2) | 3,53E-06 |
| 1473 | PE(35:4) | 4,93E-06 |  | 1511 | SM4(d43:2) | 3,51E-06 |
| 1474 | SM(d26:0) | 4,92E-06 |  | 1512 | PA(38:3) | 3,5E-06 |
| 1475 | TG(45:6) | 4,91E-06 |  | 1513 | PE(44:10) | 3,47E-06 |
| 1476 | TG(70:1) | 4,89E-06 |  | 1514 | HexCer(d30:3) | 3,45E-06 |
| 1477 | PC(32:5) | 4,88E-06 |  | 1515 | PC(O-41:8) | 3,37E-06 |
| 1478 | SM(d44:0) | 4,84E-06 |  | 1516 | PE(41:5) | 3,35E-06 |
| 1479 | TG(70:6) | 4,82E-06 |  | 1517 | TG(42:7) | 3,31E-06 |
| 1480 | PS(36:3) | 4,81E-06 |  | 1518 | TG(72:2) | 3,3E-06 |
| 1481 | LPC(21:4) | 4,7E-06 |  | 1519 | TG(41:4) | 3,26E-06 |
| 1482 | TG(39:3) | 4,65E-06 |  | 1520 | TG(O-61:3) | 3,23E-06 |

| 1521 | SM(t34:3) | 3,23E-06 |  | 1559 | PE(36:0) | 1,93E-06 |
| --- | --- | --- | --- | --- | --- | --- |
| 1522 | PC(22:0) | 3,2E-06 |  | 1560 | PE(33:0) | 1,92E-06 |
| 1523 | TG(71:2) | 3,18E-06 |  | 1561 | TG(72:4) | 1,9E-06 |
| 1524 | PC(45:6) | 3,16E-06 |  | 1562 | SM(d46:4) | 1,85E-06 |
| 1525 | TG(70:7) | 3,15E-06 |  | 1563 | TG(71:3) | 1,81E-06 |
| 1526 | PC(46:3) | 3,12E-06 |  | 1564 | LPE(O-23:1) | 1,79E-06 |
| 1527 | SM(t30:0) | 3,1E-06 |  | 1565 | PC(O-43:8) | 1,79E-06 |
| 1528 | LPE(O-22:0) | 3,06E-06 |  | 1566 | TG(72:1) | 1,78E-06 |
| 1529 | TG(67:7) | 3,05E-06 |  | 1567 | TG(52:12) | 1,75E-06 |
| 1530 | SM(d28:3) | 2,98E-06 |  | 1568 | SM4(d40:0) | 1,71E-06 |
| 1531 | SM(d46:3) | 2,95E-06 |  | 1569 | TG(O-61:4) | 1,71E-06 |
| 1532 | TG(O-64:2) | 2,94E-06 |  | 1570 | TG(71:4) | 1,68E-06 |
| 1533 | TG(69:6) | 2,9E-06 |  | 1571 | SM(t29:1) | 1,66E-06 |
| 1534 | TG(72:3) | 2,86E-06 |  | 1572 | TG(O-64:3) | 1,65E-06 |
| 1535 | PC(48:4) | 2,84E-06 |  | 1573 | TG(O-62:0) | 1,64E-06 |
| 1536 | SM(d46:1) | 2,75E-06 |  | 1574 | PE(44:4) | 1,6E-06 |
| 1537 | LPE(15:0) | 2,74E-06 |  | 1575 | DG(45:3) | 1,6E-06 |
| 1538 | TG(65:8) | 2,73E-06 |  | 1576 | PE(O-48:8) | 1,57E-06 |
| 1539 | PC(O-47:6) | 2,68E-06 |  | 1577 | PA(36:5) | 1,52E-06 |
| 1540 | PE(O-36:8) | 2,66E-06 |  | 1578 | PE(30:1) | 1,52E-06 |
| 1541 | PC(46:11) | 2,55E-06 |  | 1579 | LPC(28:2) | 1,49E-06 |
| 1542 | SM(t32:3) | 2,46E-06 |  | 1580 | PA(40:5) | 1,47E-06 |
| 1543 | SM4(t43:1) | 2,44E-06 |  | 1581 | PE(O-34:6) | 1,45E-06 |
| 1544 | TG(70:0) | 2,43E-06 |  | 1582 | TG(72:5) | 1,42E-06 |
| 1545 | LPE(24:1) | 2,4E-06 |  | 1583 | TG(73:2) | 1,37E-06 |
| 1546 | TG(O-63:1) | 2,37E-06 |  | 1584 | TG(O-64:5) | 1,37E-06 |
| 1547 | TG(O-64:1) | 2,36E-06 |  | 1585 | SM4(d42:4) | 1,34E-06 |
| 1548 | PE(39:3) | 2,35E-06 |  | 1586 | PE(O-29:1) | 1,33E-06 |
| 1549 | LPC(27:0) | 2,33E-06 |  | 1587 | SM4(d43:1) | 1,3E-06 |
| 1550 | PE(O-42:11) | 2,3E-06 |  | 1588 | TG(35:2) | 1,28E-06 |
| 1551 | TG(71:1) | 2,21E-06 |  | 1589 | TG(44:8) | 1,23E-06 |
| 1552 | SM4(d39:1) | 2,19E-06 |  | 1590 | LPC(27:3) | 1,21E-06 |
| 1553 | PE(O-43:8) | 2,15E-06 |  | 1591 | PA(34:3) | 1,2E-06 |
| 1554 | TG(43:6) | 2,1E-06 |  | 1592 | LPC(O-21:1) | 1,18E-06 |
| 1555 | TG(O-63:2) | 2,09E-06 |  | 1593 | TG(48:10) | 1,18E-06 |
| 1556 | TG(O-61:0) | 2,09E-06 |  | 1594 | TG(74:3) | 1,14E-06 |
| 1557 | TG(O-59:5) | 1,98E-06 |  | 1595 | TG(71:0) | 1,12E-06 |
| 1558 | PE(O-45:7) | 1,96E-06 |  | 1596 | TG(O-66:2) | 1,1E-06 |

| 1597 | TG(74:2) | 1,09E-06 |
| --- | --- | --- |
| 1598 | LPC(23:2) | 1,07E-06 |
| 1599 | LPC(26:5) | 1,06E-06 |
| 1600 | LPC(O-23:2) | 1,05E-06 |
| 1601 | TG(47:8) | 1,04E-06 |
| 1602 | TG(73:3) | 1,03E-06 |
| 1603 | SM4(t36:3) | 1,02E-06 |
| 1604 | SM4(d32:2) | 1,01E-06 |
| 1605 | TG(O-66:8) | 9,72E-07 |
| 1606 | TG(71:5) | 9,03E-07 |
| 1607 | PC(48:9) | 8,52E-07 |
| 1608 | TG(50:11) | 8,51E-07 |
| 1609 | PE(O-35:7) | 8,16E-07 |
| 1610 | SM4(d44:2) | 7,58E-07 |
| 1611 | TG(46:9) | 7,21E-07 |
| 1612 | TG(37:3) | 7,14E-07 |
| 1613 | LPC(28:5) | 6,81E-07 |
| 1614 | TG(O-66:3) | 6,68E-07 |
| 1615 | TG(38:5) | 6,06E-07 |
| 1616 | TG(74:4) | 5,8E-07 |
| 1617 | SM4(d44:3) | 5,71E-07 |
| 1618 | TG(72:0) | 5,07E-07 |
| 1619 | LPC(11:0) | 3,9E-07 |
| 1620 | SM4(t32:0) | 3,84E-07 |
| 1621 | TG(39:4) | 2,98E-07 |
| 1622 | LPC(25:2) | 2,64E-07 |
| 1623 | TG(36:4) | 1,74E-07 |
| 1624 | TG(72:13) | 6,46E-08 |
| 1625 | TG(38:6) | 2,6E-08 |
